# Design of machine learning-based controllers for speed control of PMSM drive

**DOI:** 10.1038/s41598-025-02396-y

**Published:** 2025-05-22

**Authors:** Ashly Mary Tom, J. L. Febin Daya

**Affiliations:** 1 School of Electrical Engineering, Vellore Institute of Technology - Chennai Campus, Chennai, 600127 India; 2 Electric Vehicles Incubation, Testing and Research Center, Vellore Institute of Technology - Chennai Campus, Chennai, 600127 India

**Keywords:** Controllers, Long short-term memory network, Machine learning (ML), Neural network, Permanent magnet synchronous motor (PMSM), Vector control., Engineering, Electrical and electronic engineering, Software

## Abstract

This study presents machine learning (ML)-based controllers for a surface permanent magnet synchronous motor (PMSM) drive system. The ML-based regression techniques like linear regression (LR), support vector machine regression (SVM), feedforward neural network (NN) and advanced NN like Long Short-Term Memory network (LSTM) are explored here in detail. This paper aims to develop an improved vector controller based on machine learning, and to investigate ML algorithms which are not yet been explored for the current control of a PMSM drive. The proposed machine learning-based control approach, which explores the influence of decoupling terms on vector control, is theoretically investigated and simulated in the vector control environment of the PMSM drive. The performance is also evaluated in real-time using the Opal-RT setup. The proposed control approach demonstrates the ability to fulfill the speed tracking requirements in the closed-loop drive system. A comparison of the simulation results between the PI controller and the suggested control algorithms validates the effectiveness of the proposed control algorithms for speed control applications. The performances of the proposed ML-based controllers improved in terms of evaluation metrics, transient peak levels and current responses, when compared to the conventional PI controller.

## Introduction

Given the growing expense of fuel and environmental problems like global warming and climate change, renewable and sustainable energy solutions are becoming essential. Because they make it easier to use green energy technology, electrical machines are becoming more and more popular^1–3^. With their unique benefits - such as good torque characteristics, low maintenance costs, fast dynamic response, high energy/mass ratio, better permanent magnet cost, better heat dissipation to the environment, high effectiveness, and long service life - Permanent Magnet Synchronous machines (PMSM) are among the most promising types of AC machines^1–9^. Therefore, PMSM machines are widely used in many different applications, such as wind power generation, servo drives, aviation, electric and hybrid vehicles (EV/HEV), and home appliances^1,2,10,11^.

Scalar control and vector control are the two most popular approaches for controlling the speed of PMSM motors. The simplicity of the control principle makes the scalar control method ideal for systems that do not require high accuracy. There are two widely used vector control methods: Field Oriented Control (FOC) and Direct Torque Control (DTC). They enhance the effectiveness and quality of PMSM speed control and are widely used because of their key advantages - simplicity in the control structure, ease of operation, reliability, and high efficiency^1,3,7,12^.

In DTC drives, accuracy depends on the flux observer, and in real-world applications, observer accuracy may significantly decrease at low speeds. The dq-axes currents in FOC drives are calculated from measured currents and rotor position, allowing accurate tracking of current commands. Furthermore, the FOC control method can regulate the semiconductor switching power loss and switching frequency better than the typical DTC controller approach, making it more popular in industrial systems. The FOC control method has become increasingly popular in high-performance AC drives during the last two decades, owing to significant advances in power electronics, computers, and microelectronics^1–3^.

The FOC control approach uses PID (proportional-integral-derivative) or PI (proportional-integral) controllers. These controllers have the advantages of simplicity, ease of construction, and practical applicability. However, because of their sensitivity to system uncertainties, these fixed-gain controllers require an exact model of the system to determine the controller gain values^5,13^. The automotive industry continues to use PI controllers in the FOC control approach, despite numerous efforts to replace or improve upon them^7,14–18^.

In comparison to PI/PID controllers, Artificial Intelligence (AI) controllers can be trained with sufficient data. These controllers are capable of performing satisfactorily well even in ill-defined models. These AI controllers include neural network-based controllers, fuzzy-based controllers, and genetic algorithm-based controllers^12^. Major drawbacks are memory space limitations, and the long time required for the training and learning procedure. These issues are taken care of using the graphical processing units (GPU)^19^.

A subset of artificial intelligence (AI), called machine learning (ML) is capable of extracting useful patterns from massive volumes of data or transforming experience into expertise. This technique uses domain knowledge rather than mathematical modeling to create and train an ML model^20,21^. The four main categories of machine learning techniques include supervised, semi-supervised, unsupervised, and reinforcement learning. The most popular approach is supervised learning, which is further classified into two categories: regression, which yields continuous output, and classification, which yields discrete output^22–24^.

The significant advantage of the regression-based approach is its capacity to perform well on minimal datasets^20^. The neural network models also have the capability to learn and adapt to complex correlation problems^12^. The speed control of electric motors can be described and modelled as a regression problem. Some of the most popular ML regression methods are linear regression (LR), decision trees, random forests, support vector machines (SVM), and neural networks (NN)^25,26^ and few of them are examined in this proposed work. Advanced neural networks or deep learning techniques such as Recurrent Neural Network (RNN), Long Short-Term Memory network (LSTM), bi-directional LSTM, and Gated Recurrent Units (GRU) are also available for regression problems^24,27,28^, and LSTM is explored in this paper. A review of the existing literature reveals that, to date, only NN models have been investigated and tested to improve the current control of a PMSM drive. This paper attempts to study other regression techniques which are not already addressed in the existing literature, for the current control application in traction drives^7,14,29,30^.

The main objectives of this paper are:

1) To develop an ML-based current controller with simpler circuitry.

2) To explore the potential of several ML regression methods to effectively manage the speed of a PMSM motor drive utilized in electric vehicles (EV).

3) To present an alternative to the conventional controller used in the industry, from the emerging field of Artificial Intelligence.

The novelties of this paper are as follows:

1) This paper proposes to use ML algorithms of different complexity levels such as LR, SVM and LSTM, in addition to existing NN models. The existing literature has explored only NN models till date in speed control application of PMSM drives and we aim to find a better and less complex ML algorithm for the same.

2) The proposed work eliminates the internal circuitry that was necessary for the conventional model to rectify the decoupling inaccuracy in the motor modelling. The proposed ML-based controllers are independent of this requirement; and outperform or at least match the performance of the conventional model. As a result, compared to the conventional model, the proposed models have less circuitry and are cost-effective.

3) To correct decoupling inaccuracy, we propose inclusion of decoupling terms to the ML-based controller inputs; while the conventional FOC technique add them at the end of the current loop through a small inner loop. This step ensures that any unexpected changes in the motor outputs, and thereby the decoupling terms, will be reflected accurately in the ML inputs and thus the control process.

4) With the above step, this paper finds the LR-based controller to be a promising alternative to the PI current controller in the motor drive, based on the statistical performance measures and test results. The LR-based controller outperforms the PI controller by 1.86% in performance parameters and responds 40% faster in the current response. The conventional controller is matched in performance by the SVM and NN-based controllers. LSTM-based controller was explored but found to be in need of more fine-tuning to satisfy the requirements.

The organization of this paper is as follows: theoretical information about PMSM drives, the standard vector control mechanism, the proposed approach, and the machine learning techniques used are discussed in Section II and Section III. Section IV shows the performance analysis of the suggested models under various criteria. Section V presents the result discussions and Section VI gives the conclusion.

## Conventional methodology

### PMSM model

The stator voltage equations of the PMSM in the synchronously rotating coordinate system (dq-axes)^11,13,29,31^ are given by

1 $$\:\left(\begin{array}{c}{v}_{sd}\\\:{v}_{sq}\end{array}\right)=\left(\begin{array}{cc}{R}_{s}+{L}_{d}\frac{d}{dt}&\:-{w}_{e}{L}_{q}\\\:{w}_{e}{L}_{d}&\:{R}_{s}+{L}_{q}\frac{d}{dt}\end{array}\right)\left(\begin{array}{c}{i}_{d}\\\:{i}_{q}\end{array}\right)+\left(\begin{array}{c}0\\\:{w}_{e}{\psi\:}_{f}\end{array}\right)$$

where *v*_***sd***_, *v*_***sq***_ are instantaneous stator voltages in dq-axes; *i*_***dq***_ are instantaneous stator currents; *R*_***s***_, *L*_***dq***_ are the resistance and inductances of the stator winding; *ω*_***e***_ is the motor speed; and *ψ*_***f***_ is the permanent magnet (rotor) flux linkage.

The electrical torque developed in the PMSM drive is represented by

2 $$\:Te={J}_{eq}\frac{d{w}_{m}}{dt}+{B}_{a}{w}_{m}\:+{T}_{L}\text{}$$

where *J*_***eq***_, *B*_***a***_ are the motor inertia and the coefficient of friction, respectively; *ω*_***m***_ is the mechanical rotational speed of the motor; *T*_***L***_, *T*_***e***_ are the load torque and the electromagnetic torque of the motor drive, respectively.

3 $$\:{T}_{e}=\frac{3P}{2}\left[{\psi\:}_{f}{i}_{q}+\left({L}_{d}-{L}_{q}\right){i}_{d}{i}_{q}\right]\text{}$$

where *P* represents the pole pairs. In case of a surface PMSM motor, *L*_***d***_ = *L*_***q***_. Hence the second term becomes zero in (3). The relation between electrical rotor angle *θ*_***e***_, and motor speeds *ω*_***m***_ and *ω*_***e***_ is given by

4 $$\:{w}_{e}={w}_{m}*P\text{}$$

5 $$\:{\theta\:}_{e}=\int\:{w}_{e}\:dt\text{}$$

### FOC control of PMSM drive

**Fig. 1:**
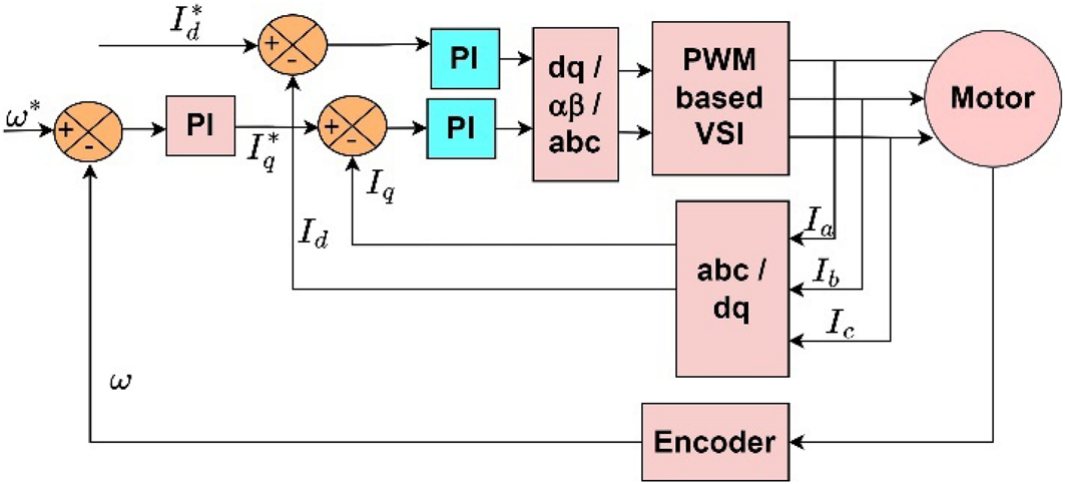
Vector control : Conventional approach.

In this method, the current through the stator is split into flux component current and torque component current along the dq-axes. Figure 1 depicts the schematic of FOC used in the PMSM drive. The conventional FOC method employs two PI controllers in the current loop, one for d-axis current and one for q-axis current.

By rearranging (2) and (3), the transfer function model for designing the controller in speed-loop is obtained as

6 $$\:{TF}_{w}=\frac{{\psi\:}_{f}.P}{\left({\:B}_{a}+s.{J}_{eq}\right)}$$

The governing equations for designing the PI controllers in the current loop are provided by rearranging (1). The compensation terms (-*w*_***e***_*L*_***q***_*i*_***q***_, *w*_***e***_*L*_***d***_*i*_***d***_, *w*_***e***_*Ψ*_***f***_) are ignored in this step. During the control process, the stator current is decoupled into flux and torque current components using the compensation terms. They are eventually combined back to construct the final current-loop control configuration, by being added to the outputs of the d-axis and q-axis PI controllers. This prevents inaccurate results brought on by decoupling^14,15,29,32,33^. In the conventional method, the gains of the PI controllers are set with the help of the Ziegler-Nicholas approach^13^.

7 $$\:{TF}_{id}=\frac{1}{\left({\:R}_{s}+s.{L}_{d}\:\right)}$$

8 $$\:{TF}_{iq}=\frac{1}{\left({\:R}_{s}+s.{L}_{q}\right)}\text{}$$

### Machine learning algorithms

In this work, the following machine learning algorithms have been used for the regression problem of speed control of a PMSM drive. Even though there are many algorithms available, only the ones listed below generated good-fit models for the problem under discussion.

#### Linear regression (LR)

The linear regression method is a widely used algorithm that represents the relationship between data by drawing a straight line through the data with the least value of Mean Square Error loss^25,26,34,35^. In (9), *y* is the output of the LR model, *x*_***i***_ is the input; *θ*_***i***_ is the weight of each *i*^th^ input, *θ*_***0***_ is the bias; and *N* is the total number of features in the dataset^20^.

9 $$\:y={\theta\:}_{0}+{\sum\:}_{i=1}^{N}{\theta\:}_{i}{x}_{i}\text{}$$

#### Support vector machine regression (SVM)

The kernel approach of SVM regression maps the independent variables onto a feature space of higher dimension. With this approach, less computing is needed and the dimensionality of the incoming data is irrelevant. The SVM regression method aims to reduce generalization error rather than training error minimization^20,34–36^.

Equation (10) is used to apply SVM regression for a dataset that is linearly separable, and (11) is utilized for a dataset that is non-linearly separable^25^. For inputs *x*_***i***_, contraction coefficient *α*_***i***_, and bias *b*, the output *y* is given by

10 $$\:y={\sum\:}_{i=1}^{N}\left({\alpha\:}_{i}-{\alpha\:}_{i}^{*}\right).〈{x}_{i},x〉+b$$

11 $$\:y={\sum\:}_{i=1}^{N}\left({\alpha\:}_{i}-{\alpha\:}_{i}^{*}\right).K\left({x}_{i},x\right)+b$$

where *K(x*,* y)* is the kernel function. For linear kernel function,

12 $$\:K\left(x,y\right)={x}^{{\prime\:}}y$$

#### Feedforward neural network (NN)

A feedforward neural network (NN) is a type of artificial neural network that only allows information to flow from the input to the output. The artificial neurons of neural networks are modeled after biological brains, and structured into three levels: input, output, and hidden layers between the two. The number of hidden layers decides the depth of the network. Each layer has a bias, while each connection between neurons is represented by a weight^25,26,35–38^.

**Fig. 2:**
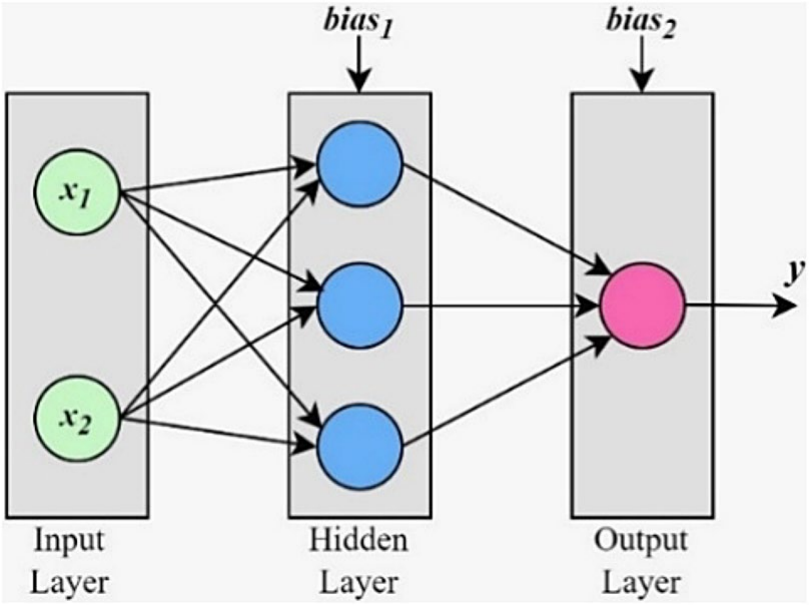
NN architecture.

**Fig. 3:**
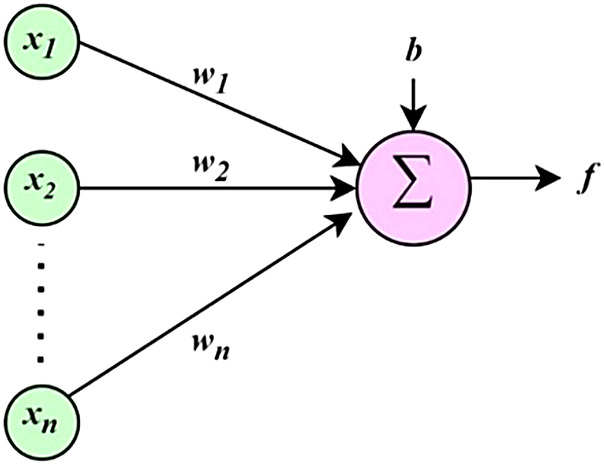
Layout of an artificial neuron.

Figure 2 shows the architecture of a NN model with two inputs and one output, and a single hidden layer with three neurons. Here *x*_***1***_, *x*_***2***_ are the inputs and *y* is the output. As seen in Fig. 3, the output of an artificial neuron is a function *f* of the sum of bias *b* and all the weights (*w*_***1***_ to *w*_***n***_) from inputs (*x*_***1***_ to *x*_***n***_)^19–21^. Activation function *f*, given by (13), is used for normalizing the output of a neuron.

13 $$\:y\:=\:\:f\left(b+{\sum\:}_{i=1}^{n}{x}_{i}{w}_{i}\right)$$

#### Long short-term memory network (LSTM)

LSTM is a variation of Recurrent Neural Network (RNN), a commonly used Deep Learning (DL) / advanced NN method for regression or forecasting purposes. One main difference between Feedforward NN and RNN is the presence of feedback loops in the latter, which helps in remembering previous information.

**Fig. 4:**
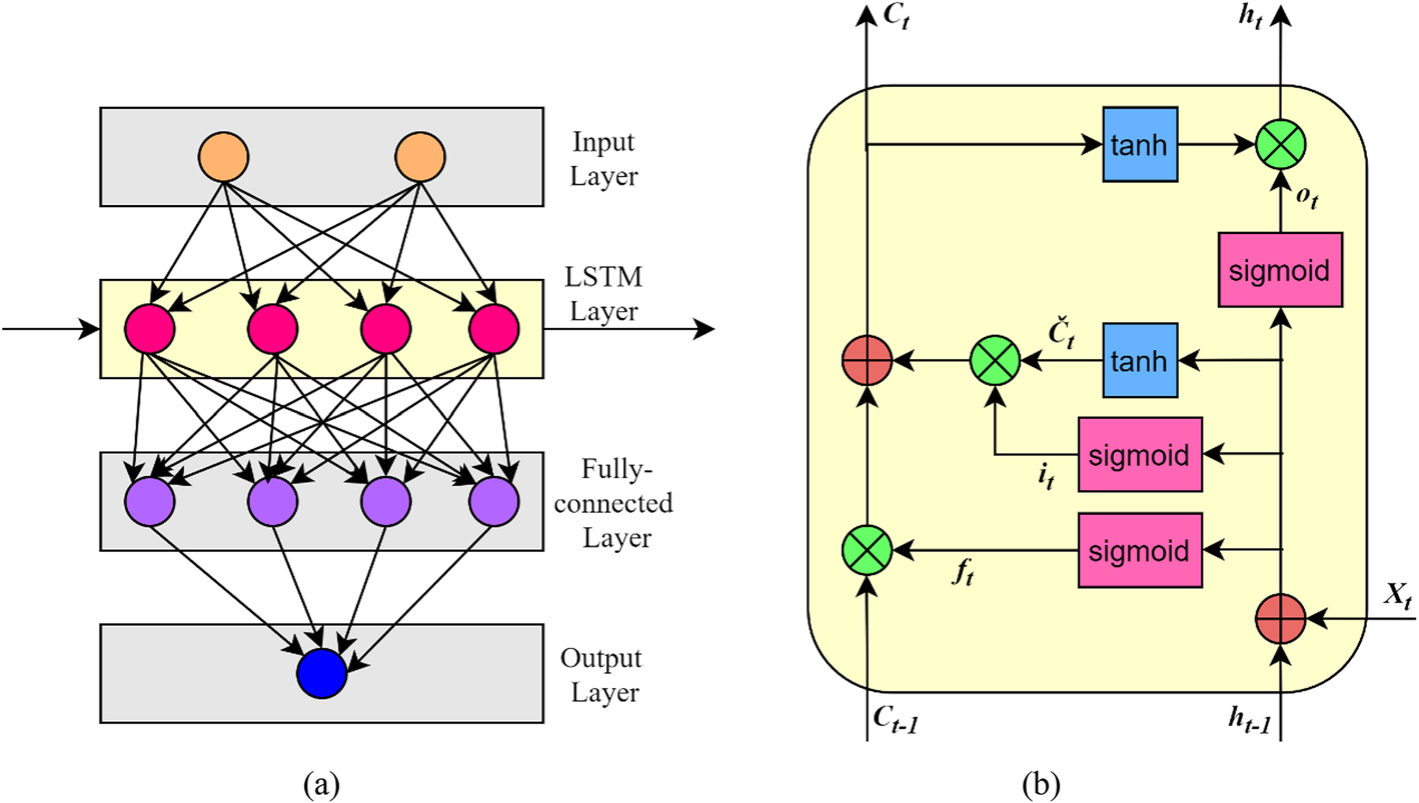
**(a)** LSTM architecture **(b)** Layout of LSTM unit.

Figure 4(a) gives the LSTM network architecture. The standard architecture includes input, hidden (consisting of LSTM and fully connected layers) and output layers. The LSTM layer is made up of a number of LSTM units connected in sequence. Figure 4(b) shows the layout of a LSTM unit.

The main components of a LSTM unit are the memory cells and the three gates (forget, input, output gates). The memory cell stores the cell state from previous time step, *C*_***t−1***_. The forget gate *f*_***t***_ decides how much information should be maintained and deleted. The input gate *i*_***t***_ decides which portion of the new information should be updated in the memory cell. The output gate *o*_***t***_ controls the information going out of the memory cell. Here, *X*_***t***_ is the input to the LSTM unit, *h*_***t***_ is the output of LSTM cell; *C*_***t***_ is the current cell state and *Č*_***t***_ is the new value to be added to the cell. The LSTM unit is mathematically expressed by (14)-(19)^25–28,39^.

14 $$\:{f}_{t}=\sigma\:\left({W}_{f}\cdot\:\left[{h}_{t-1},{X}_{t}\right]+{b}_{f}\right)$$

15 $$\:{i}_{t}=\sigma\:\left({W}_{i}\cdot\:\left[{h}_{t-1},{X}_{t}\right]+{b}_{i}\right)$$

16 $$\:\stackrel{ˇ}{{C}_{t}}=tanh\left({W}_{c}\cdot\:\left[{h}_{t-1},{X}_{t}\right]+{b}_{c}\right)$$

17 $$\:{o}_{t}=\sigma\:\left({W}_{o}\cdot\:\left[{h}_{t-1},{X}_{t}\right]+{b}_{o}\right)$$

18 $$\:{C}_{t}={f}_{t}*{C}_{t-1}+{i}_{t}*\stackrel{ˇ}{{C}_{t}}$$

19 $$\:{h}_{t}={o}_{t}*tanh\left({C}_{t}\right)$$

## Proposed methodology

### Control approach using ML

**Fig. 5:**
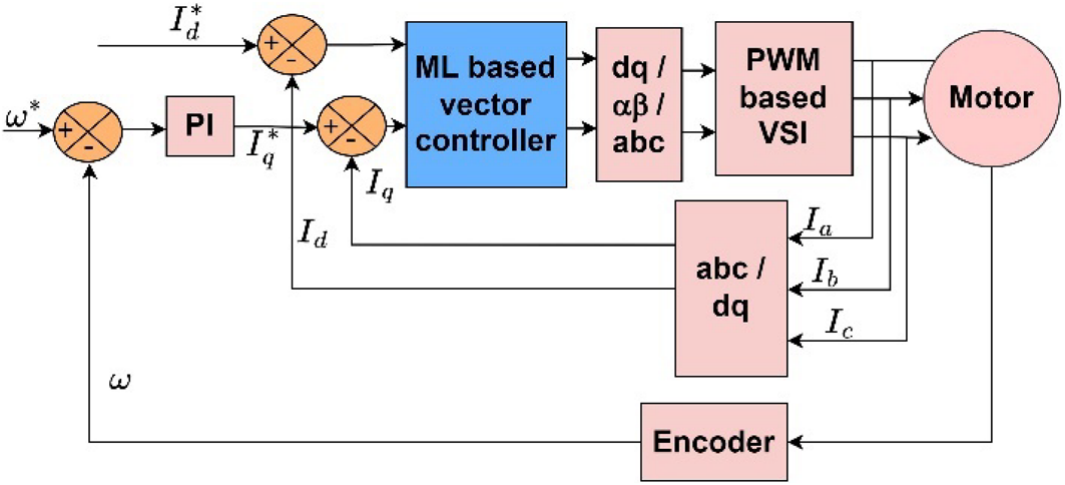
Proposed ML-based control technique.

The proposed ML-based vector control for the PMSM drive is shown in Fig. 5. The proposed approach uses an ML-based controller instead of a PI-based current controller. The speed controller in the outer loop remains the same as in the conventional approach^13,29^.

**Fig. 6:**
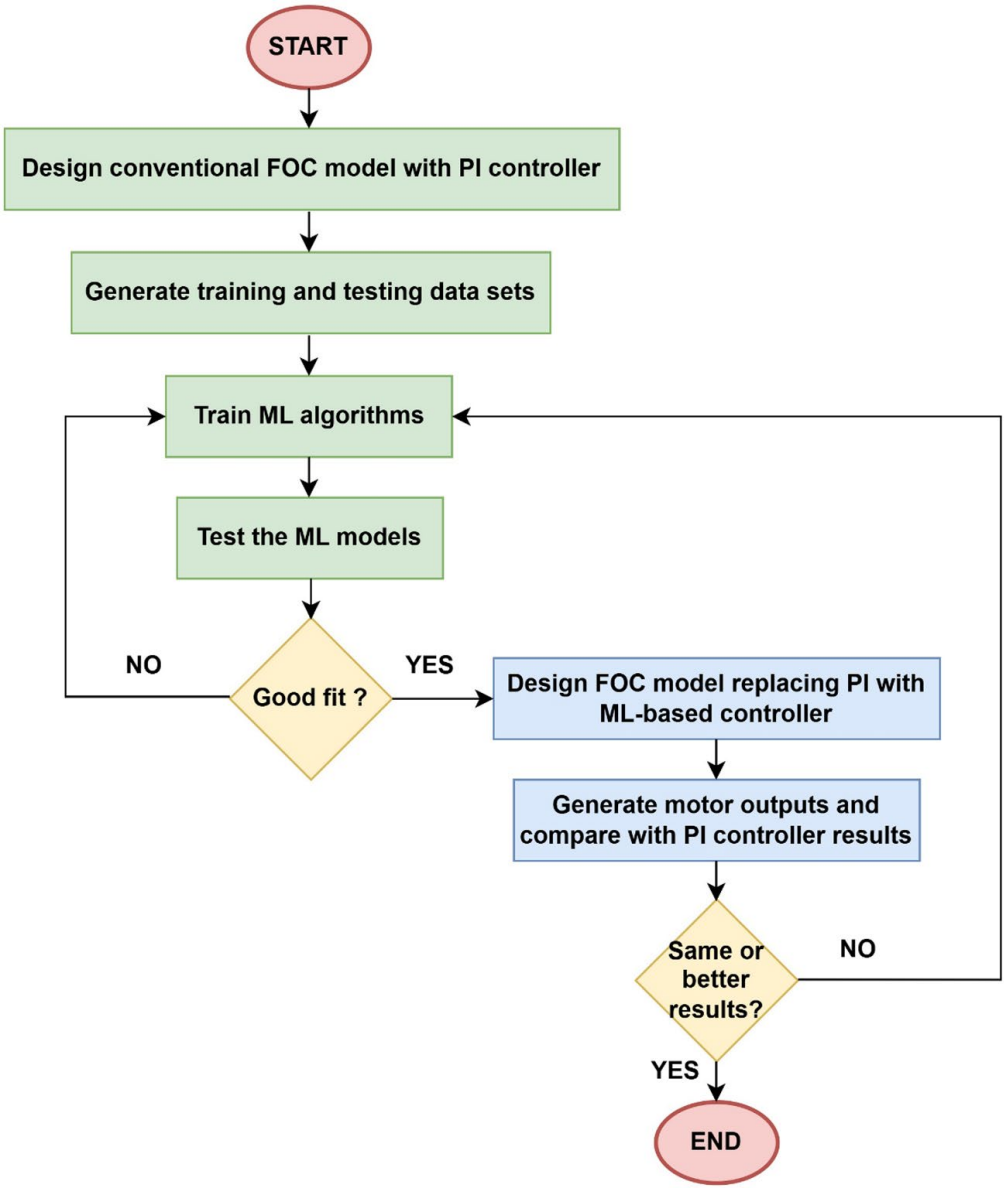
Framework of proposed ML model.

The stages of designing the proposed controller are as follows: (i) dynamic modeling (ii) ML model which is a modified version of the conventional controller approach (iii) training and testing of the ML-based controller^29^. Figure 6 shows the training and testing environment for ML models.

### Training process

The Deep Learning Toolbox™ of MATLAB is used for training and testing the ML models. The PMSM drive is operated under rated conditions with the conventional PI controller with speed variations and disturbances in load to generate a training dataset of 600,000 samples. The test dataset includes a small subset of the training data as well as new independent samples, the sample size ranging from 50,000 to 200,000.

**Fig. 7:**
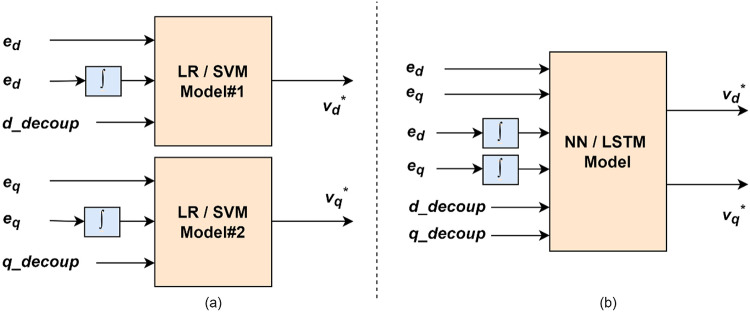
Proposed ML model layout.

Figure 7 shows the layout of the proposed ML models. The current error values, their integrals, and the compensation terms are given as input to the ML block. As LR and SVM are single-output networks, two models are required for estimating the reference voltages in the d-axis and q-axis separately. As NN and LSTM are multi-output networks, a single model takes in all the inputs and generate the two reference voltages.

**Fig. 8:**
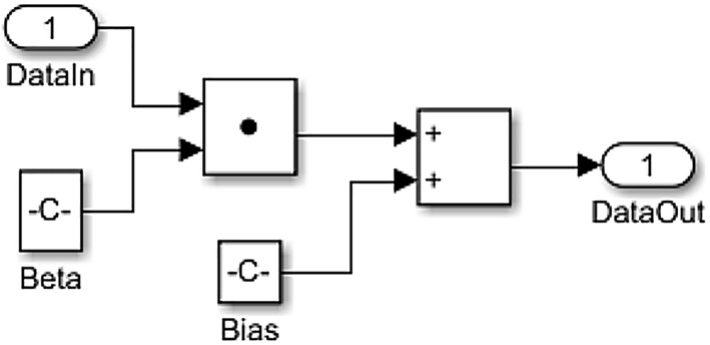
LR/SVM model layout in Simulink.

The LR and SVM blocks were developed in MATLAB/Simulink using the equations discussed in Section II – subsection C. The weights and bias values required for the modelling were obtained from the LR and SVM models developed in MATLAB Coder during training process. The layout of LR and SVM models in Simulink is given by Fig. 8. LSTM model was developed and validated in MATLAB Coder.

### Dataset

The dataset consists of 6 input variables and 2 output variables, which are displayed in Fig. 7. The differences between the desired values of the dq-axis currents and their actual values obtained from the feedback current signals (error values *e*_***d***_, *e*_***q***_) form one set of the inputs. Their integrated values (*s*_***d***_, *s*_***q***_) are the next set of inputs. The compensation or decoupling terms (*d_decoup*,* q_decoup*) are calculated using the actual dq-axis currents and taken in as the last set of inputs. The output variables are dq-axis reference voltages (*v*_d_^*^, *v*_q_^*^), which are used in the inverter pulse generation process. The relation between these input and output variables are described in detail in Section II.

20 $$\:{s}_{d}=\int\:{e}_{d}\:dt\text{}$$

21 $$\:{s}_{q}=\int\:{e}_{q}\:dt\text{}$$

As single-output models, linear regression and support vector machine regression require two distinct models for each of the two outputs. The d-axis input variables are given to one LR/SVM model and d-axis reference voltage is obtained. Similarly, the q-axis input variables are given to a second LR/SVM model and q-axis reference voltage is obtained (Fig. 7(a)).

Since NN/LSTM model can have multiple outputs, only one NN/LSTM model is needed to produce the two output variables. All the 6 input variables are given into the NN and LSTM-based controllers and the two dq-axis reference voltages are obtained (Fig. 7(b)).

By using error values and their integrated values, the aim is to prepare an ML-based model that mimics the PI controller, but in a better way. The difference is in how the compensation terms are placed. The PI controller ignores these terms and they must be added externally to the controller outputs, to avoid decoupling inaccuracy in the motor drive. The proposed ML-based models avoid this external addition process by including the decoupling terms in the initial stage itself. The complexity of the control algorithm can be reduced using this approach. The trained ML controller can simply replace the PI current controllers and the feedback of the decoupling terms, in a PMSM drive.

### Training and testing data

The training data as well as the testing data are obtained from the simulated model of a PI-controller-driven PMSM drive. The error values and the compensation or decoupling terms (4 of the input variables) are extracted directly from the model while the integrated values of the errors (2 of the input variables) are calculated separately during the data collection process. In the process of gathering the training and testing dataset, the two output variables are taken out of the data point where the compensation terms are applied.

The motor drive is run through rated conditions, variable speeds, and load disturbances, which generates a dataset of 600,000 samples for training purposes. Different test datasets are used for each test situation (variable speed, variable load, constant load and speed, etc.) and all test sets are extracted in the same way the training data is collected. Each test dataset includes a subset of the training data as well as new independent samples, the test sample size hence ranging from 50,000 to 200,000.

The same datasets are used for training and testing all the ML-based models. The data normalization is done by setting the training command accordingly in the MATLAB Coder. Each ML algorithm is prepared for training as mentioned in subsections E to G. Table 1 gives the tuned parameters for each ML model. After offline training for “sequence-to-sequence” regression, a test dataset is used to independently evaluate the ML-based controllers. Following this validation, the trained ML-based controllers are incorporated into the motor drive model, enabling online implementation for real-time system operation.

### Development of LR-based model

The function *fitrlinear* is used for training the LR regression model in MATLAB. The parameters configured in the training process are *Learner*, *Solver*, *Regularization*, and *Lambda*.

The *Learner* is a linear regression model type that can be set as Linear regression via ordinary least squares (leastsquares), or Support vector machine regression (svm). The *Solver* or objective function minimization technique can be Stochastic gradient descent (SGD), Average stochastic gradient descent (ASGD), Dual SGD, Broyden-Fletcher-Goldfarb-Shanno quasi-Newton algorithm (BFGS), Limited-memory BFGS (LBFGS), or Sparse Reconstruction by Separable Approximation (SpaRSA) method. *Regularization* or complexity penalty type can be set as Lasso or Ridge. *Lambda* is also known as the Regularization term strength and is calculated as the inverse of the training sample size.

After experimenting with different combinations for Learner-Solver-Regularization, the optimum combination is found to be a combination of SpaRSA and LBFGS solvers, least squares learner, lasso and ridge regularizations.

### Development of SVM-based model

SVM regression models are trained using the function *fitrsvm* in MATLAB. Some of the important parameters for SVM model training are *Solver*,* Kernel function*, and *Kernel scale*.

The *Kernel function* can be a Gaussian, Linear, Radial Basis Function (RBF) or Polynomial. The value of the *Kernel scale* is set to 1, or ‘auto’. In the latter case, the software uses a subsampling procedure to select the value. The *Solver* is the optimization routine. It can be Iterative Single Data Algorithm (ISDA), Sequential Minimal Optimization (SMO), or L1 Quadratic Programming (L1QP).

Different combinations for the above parameters are iterated and the SMO is selected as ‘Solver’. The linear kernel function of scale 1 is found to be optimal.

### Development of NN-based model

To train a simple neural network in MATLAB, the *train* function is used with the following parameters specified:

- *Number of hidden layers* and *Size of each hidden layer*,
- *Activation function* of each layer,
- *Training algorithm*.

A trial-and-error method is followed to select the *number* and *size* of the hidden layers, starting from the minimum values. A satisfactory result was obtained at 1 hidden layer, with 2 neurons.

Hyperbolic tangent sigmoid transfer function and linear function, respectively, work as the hidden layer and output layer *activation functions*.

Levenberg-Marquardt, Bayesian Regulation, Gradient descent, and Resilient back propagation (RPROP) algorithms are some of the *training algorithms* for neural networks in MATLAB, among which the Levenberg-Marquardt algorithm is the fastest and most popular.

### Development of LSTM-based model

The LSTM model developed in MATLAB has the following training parameters specified:

- *Number of hidden units*,
- *Types of layers*,
- *Gradient threshold*.

The layers are defined in the following order: input sequence layer, lstm layer, fully connected layers and output regression layer. The solver is set to ‘adam’ (Adam optimizer), with an initial learning rate of 0.01, a gradient threshold of infinity with the clipping method ‘global-l2norm’. A trial-and-error method is followed to select the number hidden units in the lstm layer, and the number of fully-connected layers. Based on the capacity of the work station used, they were finalized to 90 and 2, respectively.

**Table 1:** Hyperparameter settings for ML models.

ML model	Hyperparameter	Selection
LR	Learner	Leastsquares
	Solver	SparSA + LBFGS
	Regularization	lasso + ridge
	Lambda	1 / (Training sample size)
SVM	Kernel function	Linear
	Kernel scale	1
	Solver	SMO
NN	No. of hidden layers	1
	Size of hidden layers	2
	Activation function	Sigmoid function (hidden layer), linear function (output layer)
	Training algorithm	Levenberg-Marquardt algorithm
LSTM	No. of hidden units	90
	No. of fully connected layers	2
	Solver	adam
	Gradient Threshold method	global-l2norm

### Evaluation metrics

Mean absolute error (MAE), root mean square error (RMSE), and symmetric mean absolute percentage error (SMAPE) are the metrics used to assess the performance of the trained ML algorithms. These are typical statistical measures for evaluating the effectiveness of time-series based models^27,28,39–46^.

22 $$\:MAE=\frac{1}{N}{\sum\:}_{i=1}^{N}\left|\left({\stackrel{-}{y}}_{i}-{y}_{i}\right)\right|$$

23 $$\:RMSE=\:\sqrt{\frac{1}{N}{\sum\:}_{i=1}^{N}{\left({\stackrel{-}{y}}_{i}-{y}_{i}\right)}^{2}}$$

24 $$\:SMAPE=\frac{100\%}{N}{\sum\:}_{i=1}^{N}\frac{\left|\left(\stackrel{-}{{y}_{i}}-{y}_{i}\right)\right|}{\left(\left|\stackrel{-}{{y}_{i}}\right|+\left|{y}_{i}\right|\right)/2}$$

where *N* is the test sample size; *ȳ*_***i***_ is the estimated value and *y*_***i***_ is the actual value.

## Performance evaluation of controllers

The conventional model of the speed control system is developed using a MATLAB/Simulink environment to generate the training data. The sampling time is fixed at 1 µsec. The performance comparison of the conventional and proposed methods is conducted using MATLAB Coder. Table 2 gives the PMSM drive parameters^9,13^. The testing of the trained ML models is detailed in subsections A to E^9,13,29^.

**Table 2:** Motor parameters for simulation.

Parameter	Value
Resistance (R)	2.85 Ω
Stator inductances (L_d_, L_q_)	0.005 H
Coefficient of friction (B_a_)	1.0 e-4 Nm-s
Motor inertia (J_eq_)	8.0 e-4 kg-m^2^
Pole pairs (P)	4
Rotor flux linkage (Ψ_f_)	0.1548 Wb-turms
Rated speed (ω_m_)	300 rad/s

### Current control evaluation

**Fig. 9:**
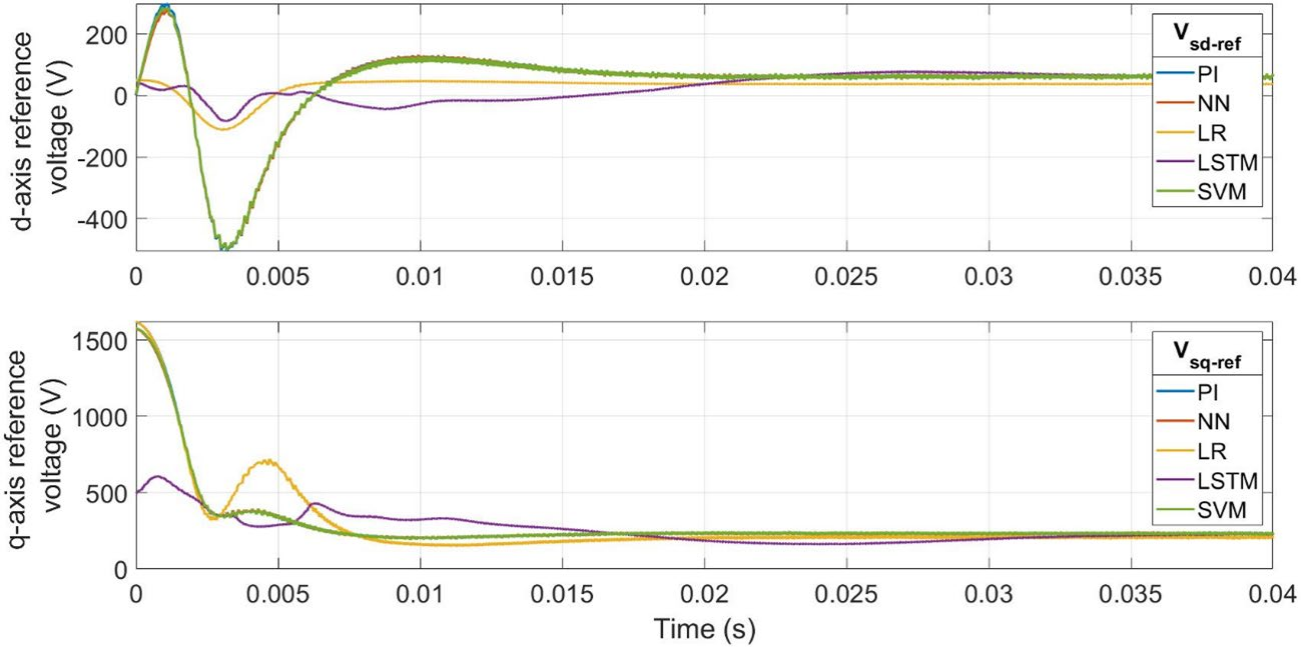
ML-based vs. conventional controller outputs : dq-axis reference voltages **(a)** ***v***_***sd-ref***_ **(b)** *v*_*sq-ref*_.

The d- and q-axis currents are studied for the evaluation of current-loop controllers while keeping a constant speed (300 rad/s) and a constant load torque (5 N.m) for the motor^9,13^. The reference voltage outputs of the proposed ML models are compared to the conventional PI controller outputs in Fig. 9. The proposed ML-based controllers exhibit reduced transient peaks in the outputs of the controller. The LR-based controller has the least maximum transient peak value for d-axis reference voltage and settles faster than the other controllers. The q-axis reference voltage waveforms of all controllers, except the LR-based controller, settle similarly to those of the PI controller.

**Fig. 10:**
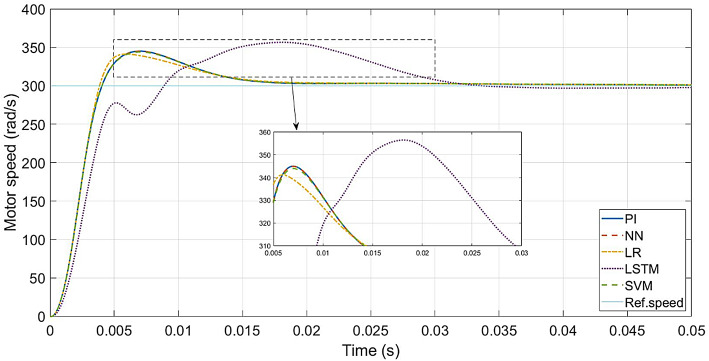
ML-based vs. conventional controllers: speed response of the drive.

**Fig. 11:**
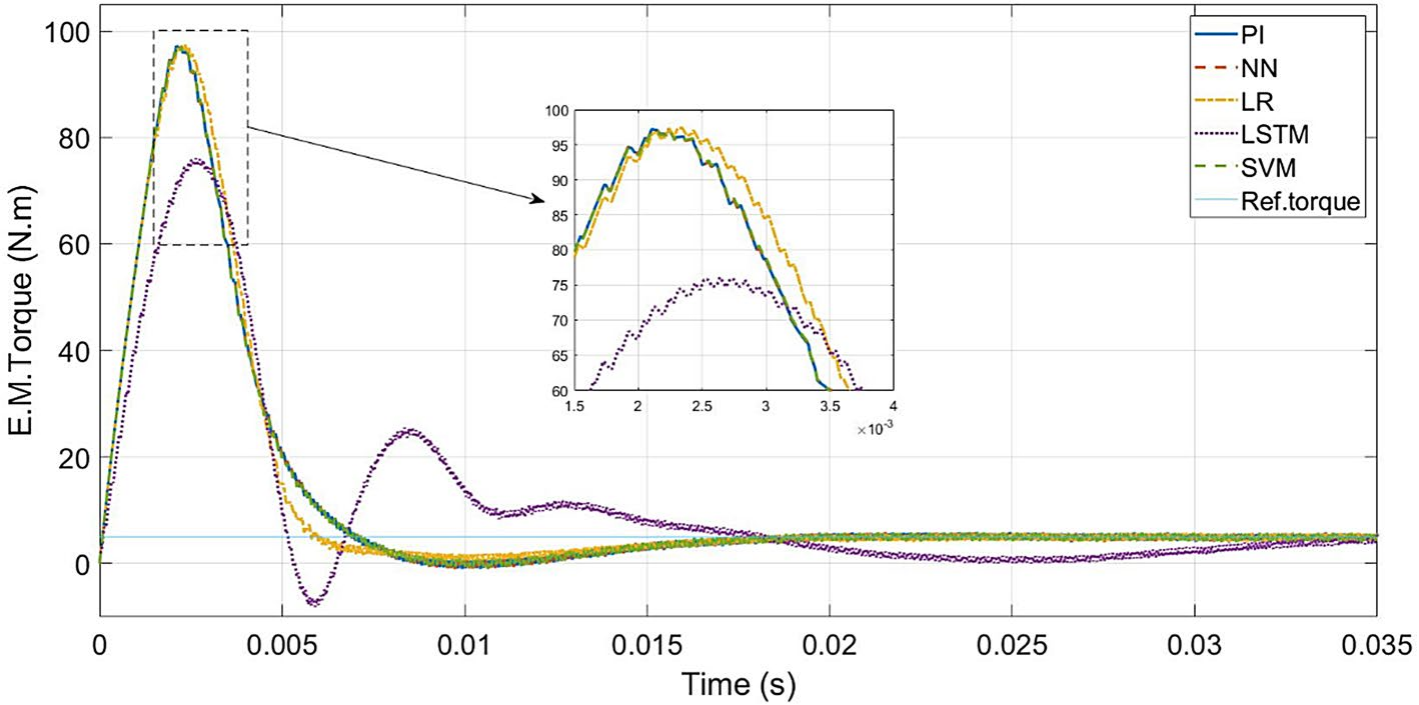
ML-based vs. conventional controllers: Torque response of the drive.

**Fig. 12:**
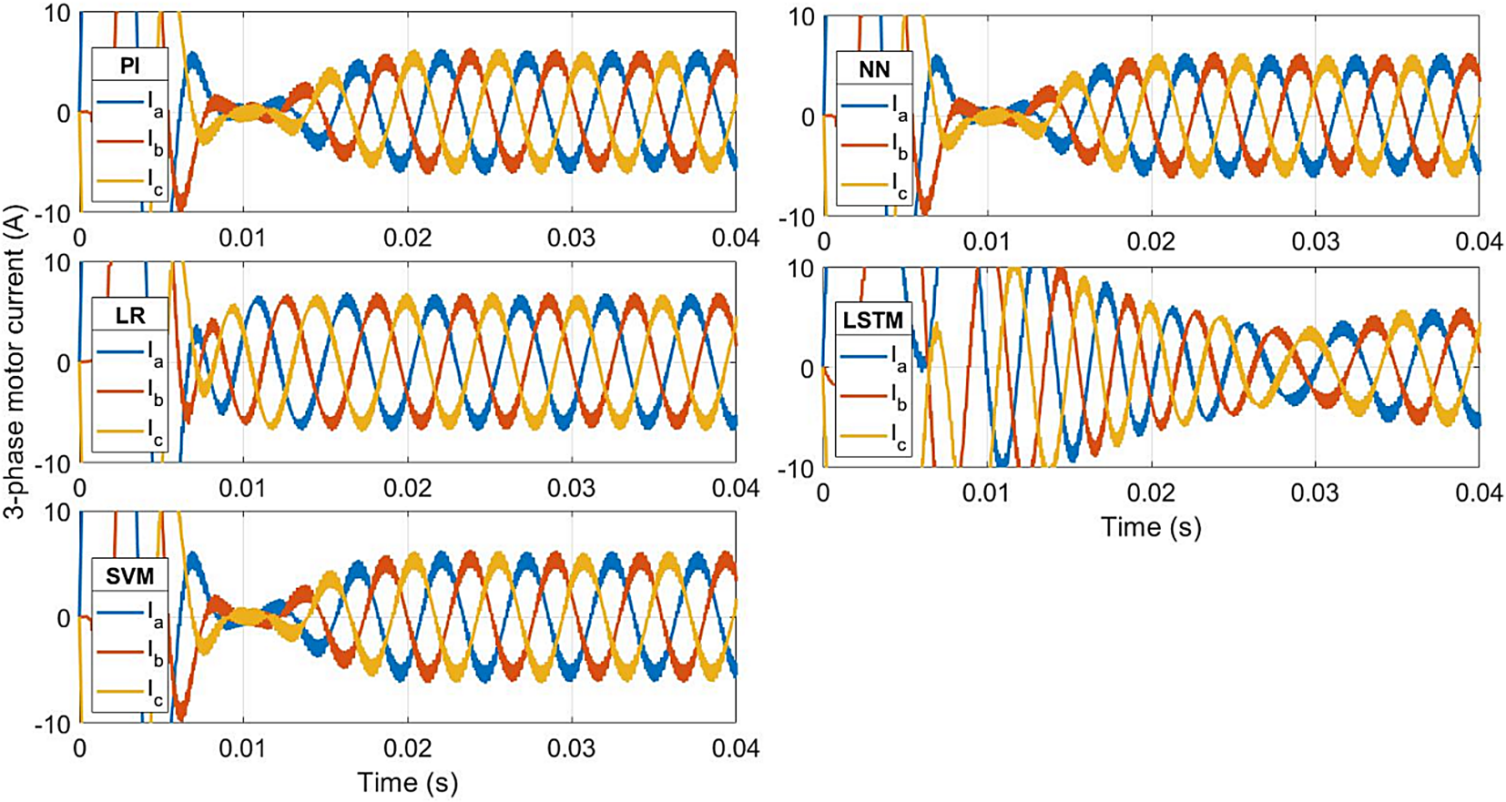
ML-based vs. conventional controllers: 3-phase motor current response.

**Fig. 13:**
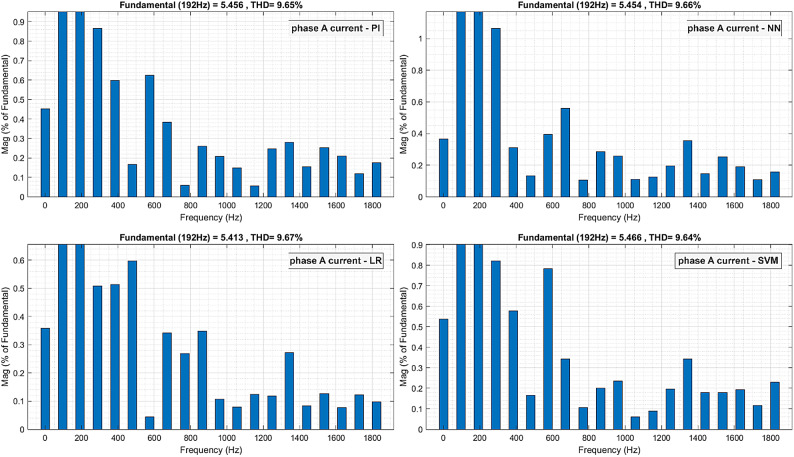
ML-based vs. conventional controllers: THD analysis of the current responses.

The performance of the conventional and ML-based controllers under rated conditions are given in Figs. 10, 11, 12 and 13. The speed response of the PI controller, as depicted in Fig. 10, achieves the desired speed with a settling time of 0.018s and a peak overshoot of 1.15%. In contrast, the LR-based controller exhibits a 1.138% peak overshoot, the SVM-based controller has a 1.147% peak overshoot, and the NN-based controller has a 1.149% peak overshoot for the same settling time. The LSTM-based controller has a 1.189% peak overshoot with 0.0325s as settling time (Table 3).

**Table 3:** Comparison of speed response under rated conditions (Fig. 10).

Description	PI	LR	SVM	NN	LSTM
Peak overshoot (%)	1.15	1.138	1.147	1.149	1.189
Settling time (s)	0.018	0.018	0.018	0.018	0.0325

For PI controller and LR, SVM, NN-based controllers, a maximum torque of 97 Nm is attained during the transient state and then settles to the rated value (5 N.m) at 0.018s. The LSTM-based controller reaches 75 Nm during transient state and settles to 5 Nm at 0.0325s. The torque response is shown in Fig. 11.

The current responses for the conventional controller and ML-based controller are shown in Fig. 12. The rated current is achieved at 0.011s with the LR-based controller, at 0.035s with LSTM-based controller, and at 0.018s with the other controllers. The transient state peak value of the LR-based controller is 86 A, 70 A for LSTM-based controller and 90 A for the other controllers.

Total Harmonic Distortion (THD) of one phase of the motor currents, for the PI controller and proposed controllers (LR, SVM, NN), were obtained by Fast Fourier Transform (FFT) analysis and are shown in Fig. 13^47^. The proposed controllers give the same THD of 9.6% as the conventional controller at the steady-state conditions.

**Table 4:** Comparison of current control performance (Fig. 14).

Settling time (s)	PI	LR	SVM	NN	LSTM
For Id	0.0085	0.02	0. 0085	0.0085	0.034
For Iq	0.018	0. 018	0. 018	0. 018	0.033

**Fig. 14:**
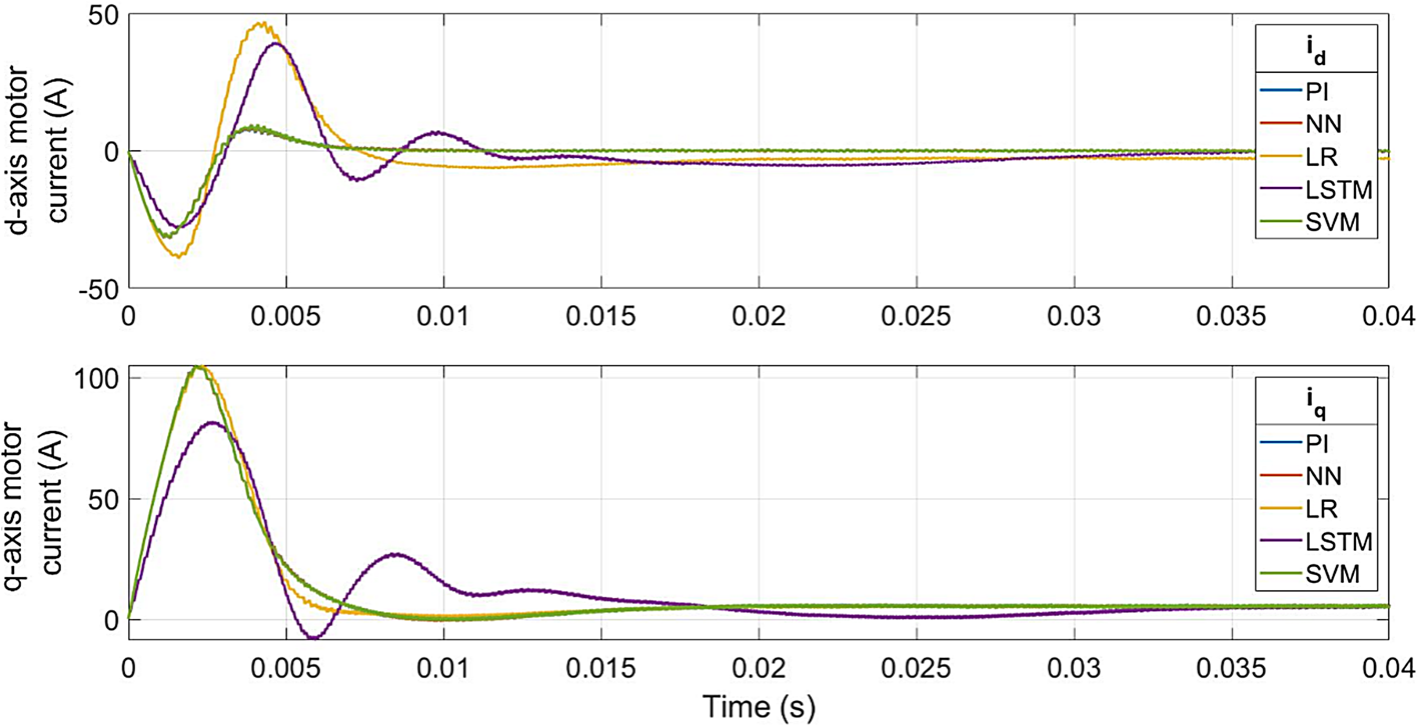
Conventional vs ML-based controllers : dq-axis currents (i_d_, i_q_) of PMSM drive.

Fig. 14 gives a comparison of the dq-axis currents of the conventional and proposed models. At 0.0085s, the d-axis current of a PI controller settles to 0 A, while the q-axis current settles to 5 A, at 0.018s. The SVM-based and NN-based controllers reach the same current values at the same time as the PI controller. The LR-based controller takes 0.02s for the d-axis current to settle at -3 A and 0.018s for the settling of the q-axis current at 5 A. For LSTM-based controller, the dq-axis currents settle at 0.034s and 0.033s respectively (Table 4).

**Fig. 15:**
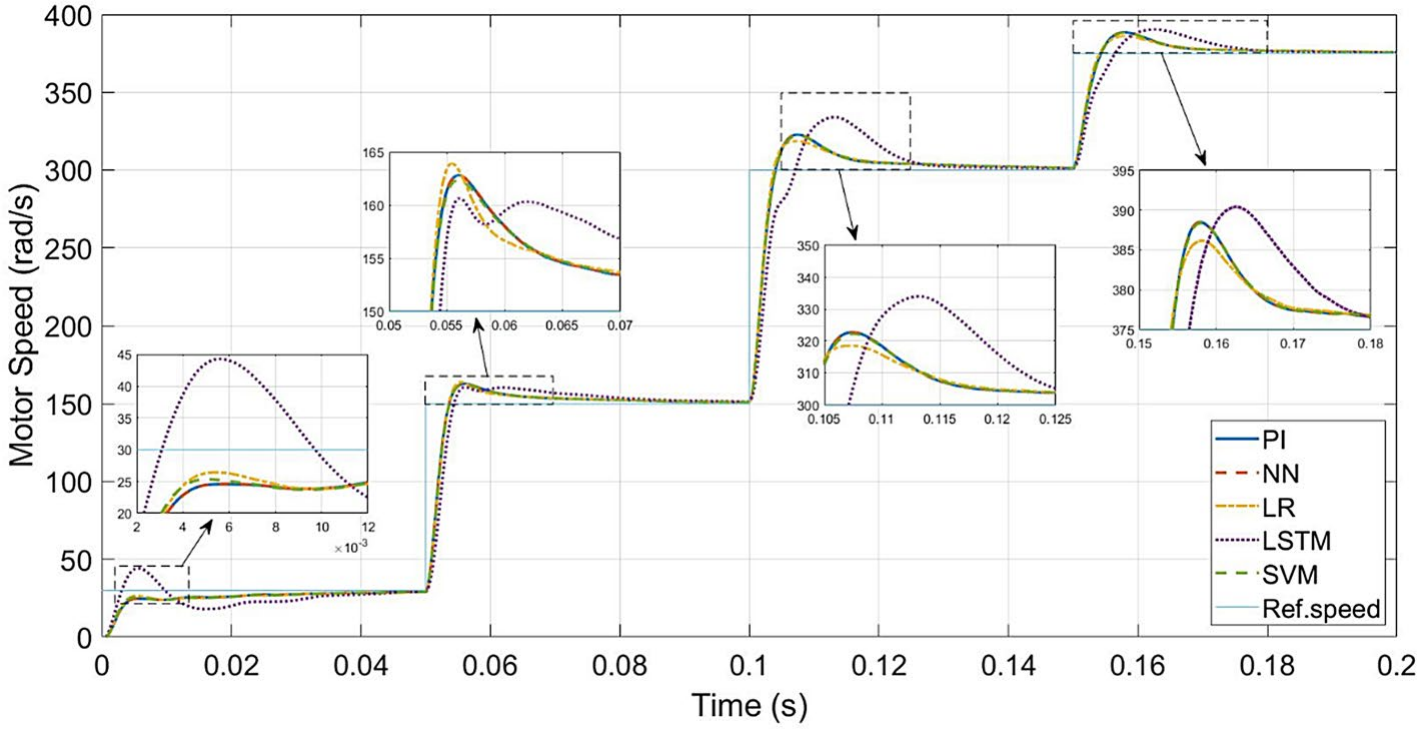
ML-based vs. conventional controllers: Speed response for speed variations.

**Fig. 16:**
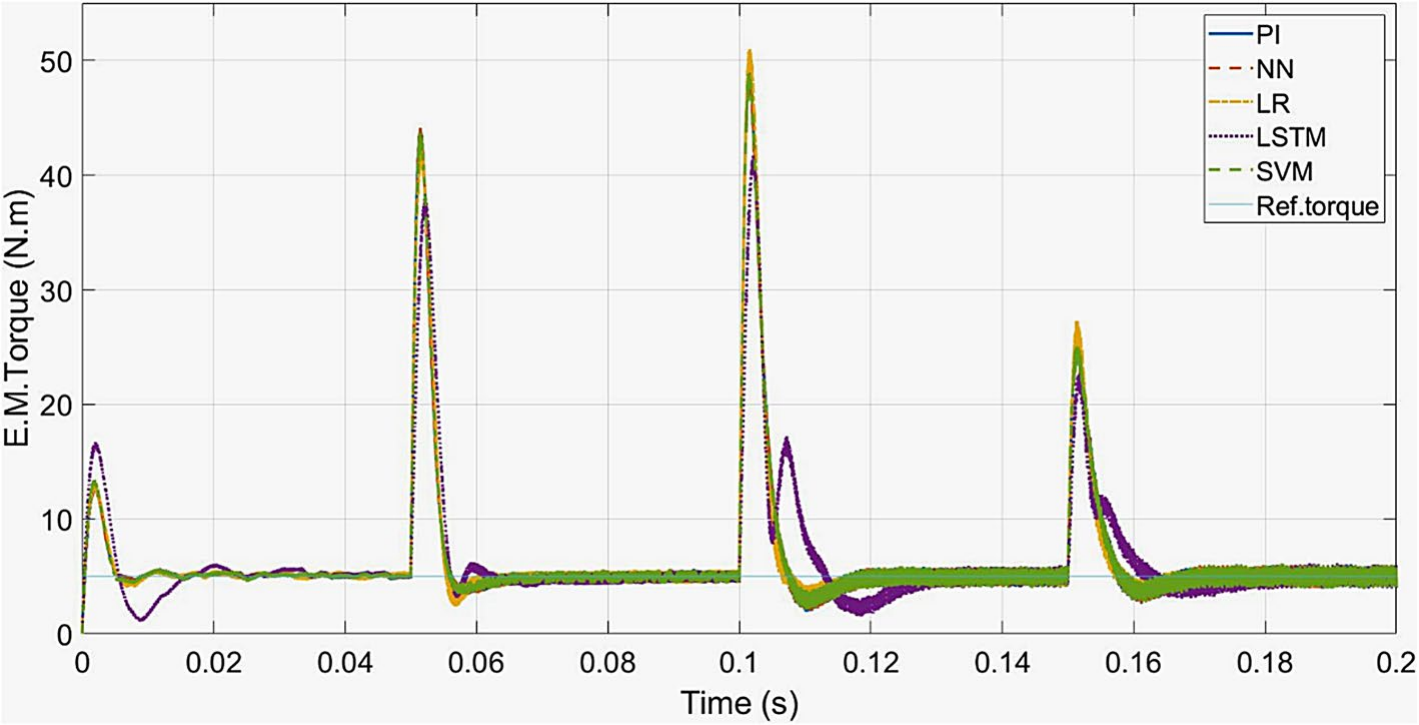
ML-based vs. conventional controllers: Torque response for speed variations.

### Speed control evaluation

To evaluate the effectiveness of the controllers, speed tracking of the PMSM is carried out. The desired speed values are set as 30–150–300–375 rad/s, applied at the time intervals of 0–0.05–0.1–0.15 s, respectively. Figure 15 gives a comparison of speed responses of all the controllers.

Up to 150 rad/s (50% of rated speed), the PI controller and the LR, SVM and NN-based controllers respond similarly, though the LR-based controller has a slightly higher transient speed peak. LSTM-based controller has speed overshoot compared to other controllers.

Above 150 rad/s (above 50% and up to 125% of rated speed), the responses of the PI controller and the LR, SVM and NN-based controllers are similar. The LR-based controller has the least transient peak at higher speeds. Above rated speed, LSTM-based controller has the highest transient peak and needs more settling time.

**Fig. 17:**
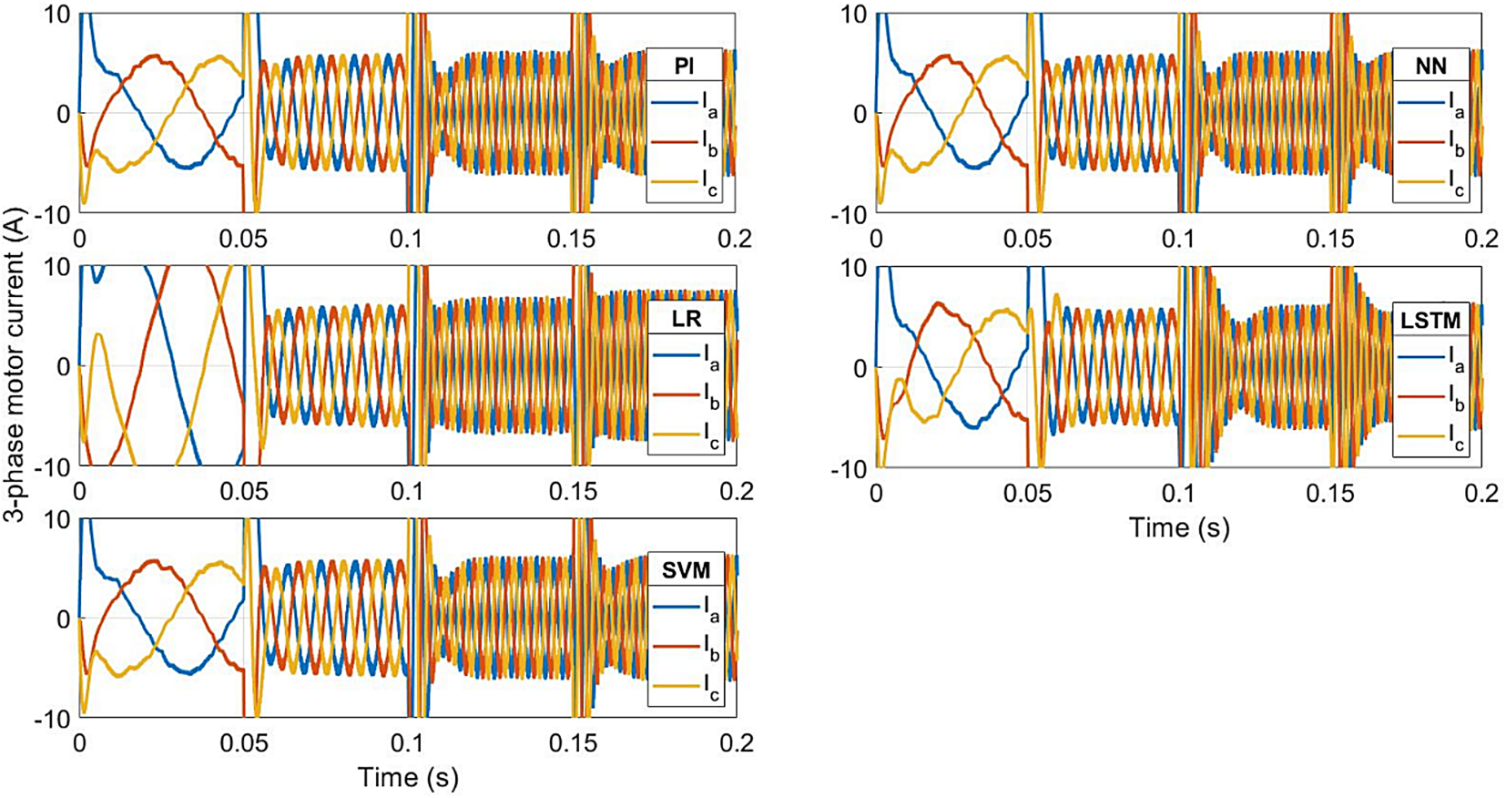
Conventional vs ML-based controllers : Current response for change in speed.

As seen from Fig. 16 to 17, the motor 3-phase currents and torque change at the same instant as the speed changes. The LSTM model has the least transient peak for torque response at speeds above 10% of the rated speed (< 30 rad/s). The LR model has the least transient peak for torque response at low speeds (30 rad/s), and the maximum transient peak in torque response at speeds above half the rated speed (> 150 rad/s). The current responses of all the controllers are similar to those of the conventional model.

### Impact of load disturbance

**Fig. 18:**
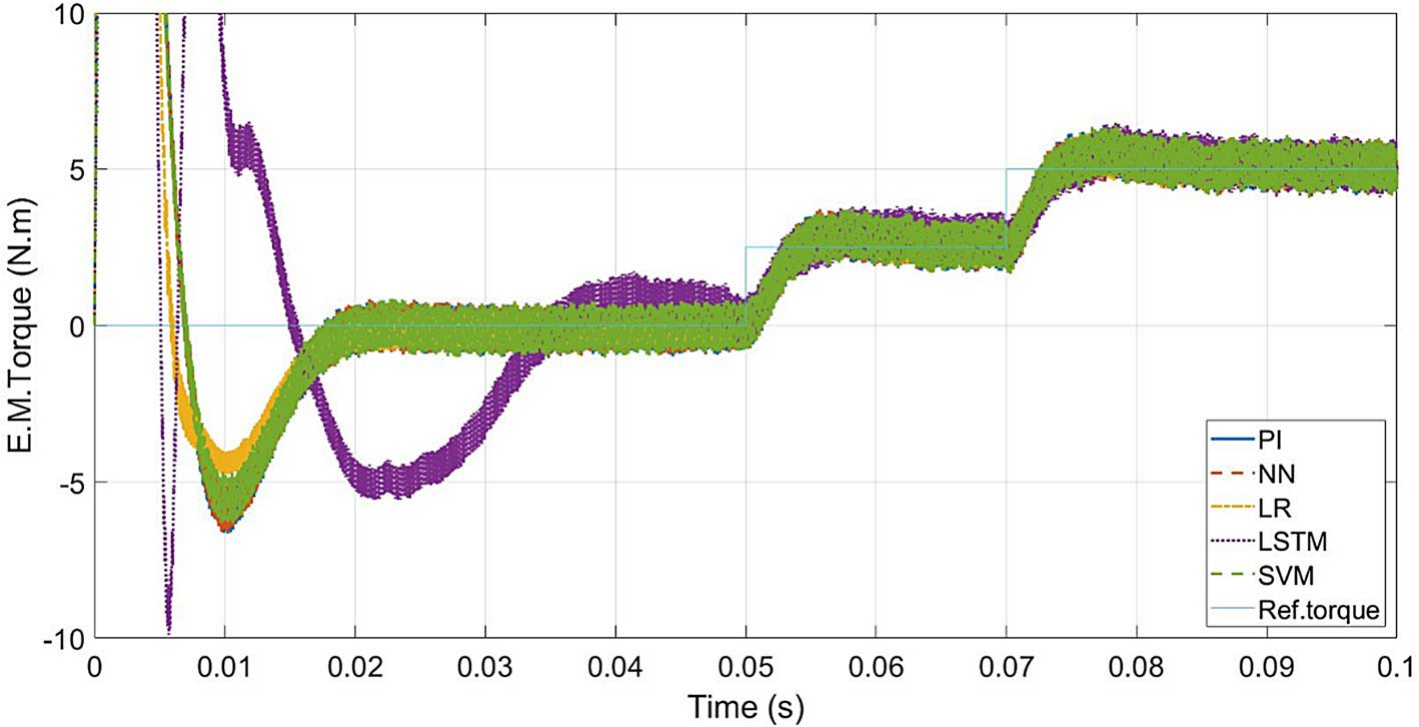
ML-based vs. conventional controllers: Torque response for load variations.

**Fig. 19:**
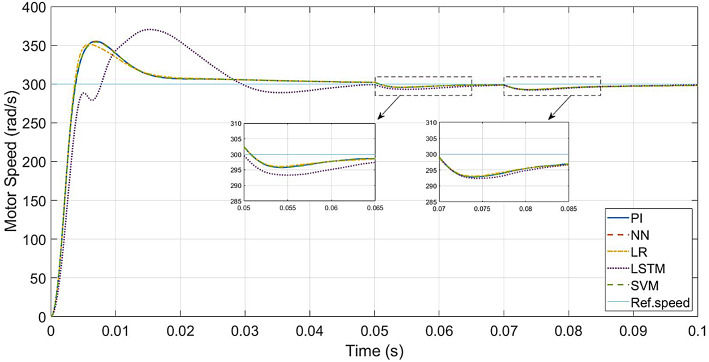
ML-based vs. conventional controllers: Speed response for load variations.

The motor is started at no load and at time step of 0.05s, load of 2.5 Nm is applied. The load is increased by another 2.5 Nm at a time step of 0.07s. Figures 18 and 19 provides a comparative performance of the torque and speed responses using both PI and ML-based controllers. The machine parameters are same as in Table 2, except for the load. Figure 18 demonstrates how the torque responses of drives with ML-based and PI controllers reflect the added load. Figure 19 illustrates how speed decreases when a load is applied, yet speed is rapidly recovered by the controllers.

### Robustness of speed loop controller

**Fig. 20:**
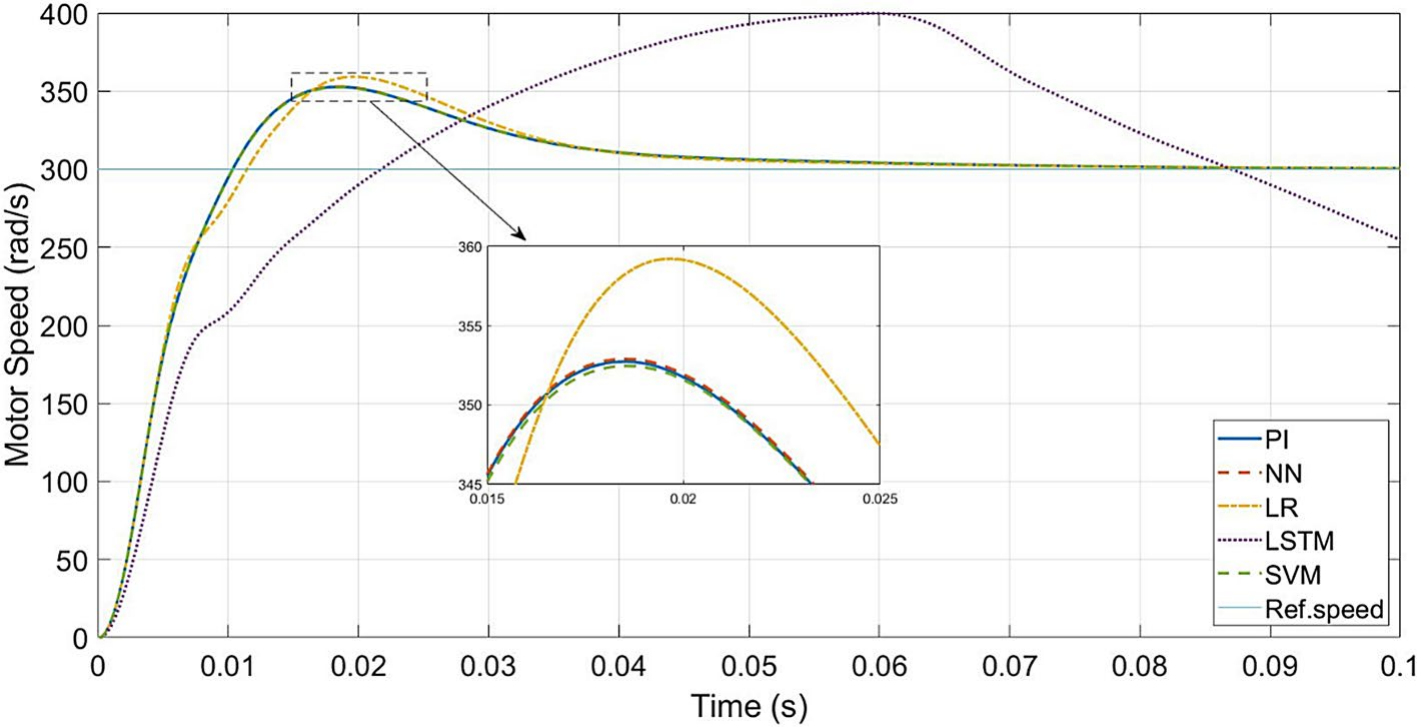
Conventional vs. ML-based controllers : Speed response for larger *J*_*eq*_ and *B*_*a*_.

In real-world situations, the motor inertia and friction factor can alter depending on the load on the EV and the state of the road. The output of the speed loop controller will be affected by this situation. As a case study, twice the friction factor and triple the inertia are considered, while the other conditions are the same as in Table 2. Figure 20 gives a comparison of the speed responses of the conventional and the ML-based vector-control approaches.

In comparison to Fig. 10, the speed responses of all the controllers, except LSTM-based controller, reach the rated speed at 0.05s. This is because of the higher inertia and friction factors. The LR-based controller has more transient peak in speed response compared to the other controllers, while the SVM-based controller has the least transient peak. The LSTM-based controller failed to settle within the same range as the other controllers.

**Table 5:** Evaluation metrics for speed response.

	Metrics	PI	LR	SVM	NN	LSTM
Rated conditions	MAE	10.9072	**10.8093**	10.9081	10.9096	19.9737
	RMSE	40.6125	**40.4614**	40.5824	40.6145	51.0321
	SMAPE	5.0976	**5.0724**	5.0998	5.0987	8.4641
Speed variations	MAE	7.5233	**7.3834**	7.5518	7.5232	10.6317
	RMSE	18.2025	**18.0162**	18.2203	18.2052	21.8243
	SMAPE	7.9562	**7.8796**	7.9181	7.9581	11.1613
Load disturbances	MAE	13.7196	**13.6210**	13.7124	13.7269	21.6626
	RMSE	40.3968	**40.2008**	40.3591	40.4021	50.9743
	SMAPE	5.9003	**5.8770**	5.8998	5.9030	8.7705

### Evaluation metrics

**Fig. 21:**
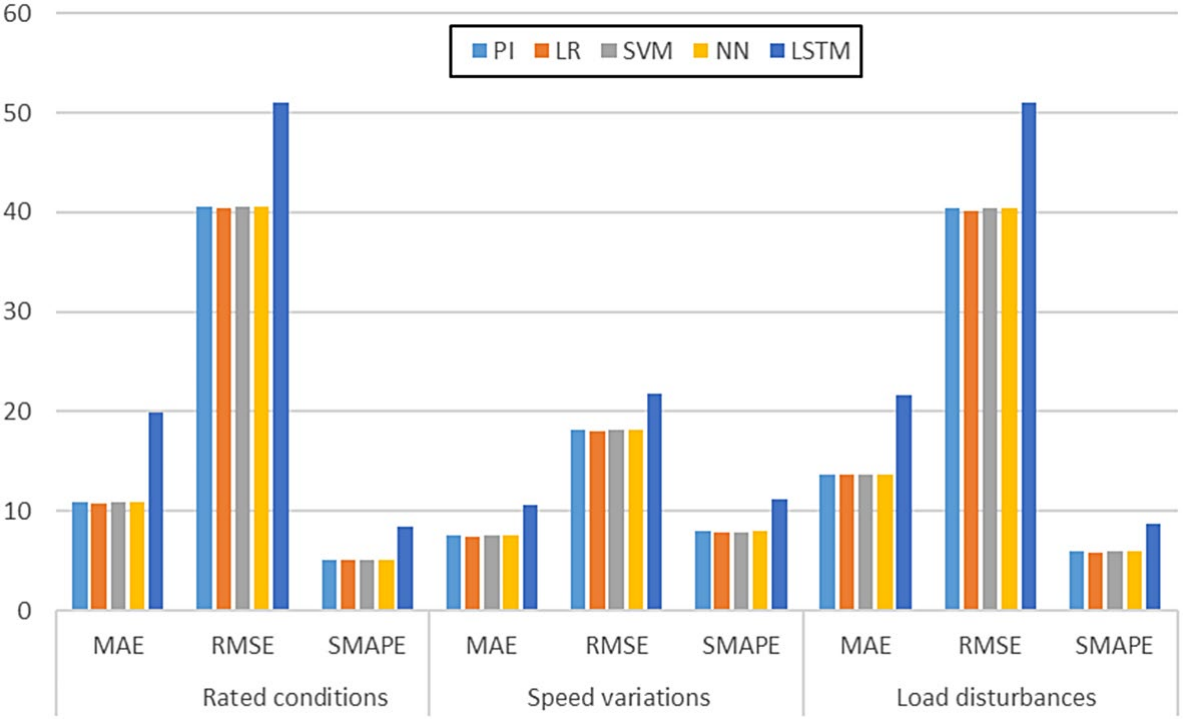
Evaluation metrics for speed response – Table 5.

The performance metrics for different test conditions such as rated speed condition, step speed change and step load change are shown in Table 5 and Fig. 21. In each of the three test situations, the LR-based controller provides the lowest values of all the errors. The least error in each set is highlighted in bold. Lesser error values of MAE, RMSE and SMAPE imply better performance of the evaluated model.

### Real-time results

**Fig. 22:**
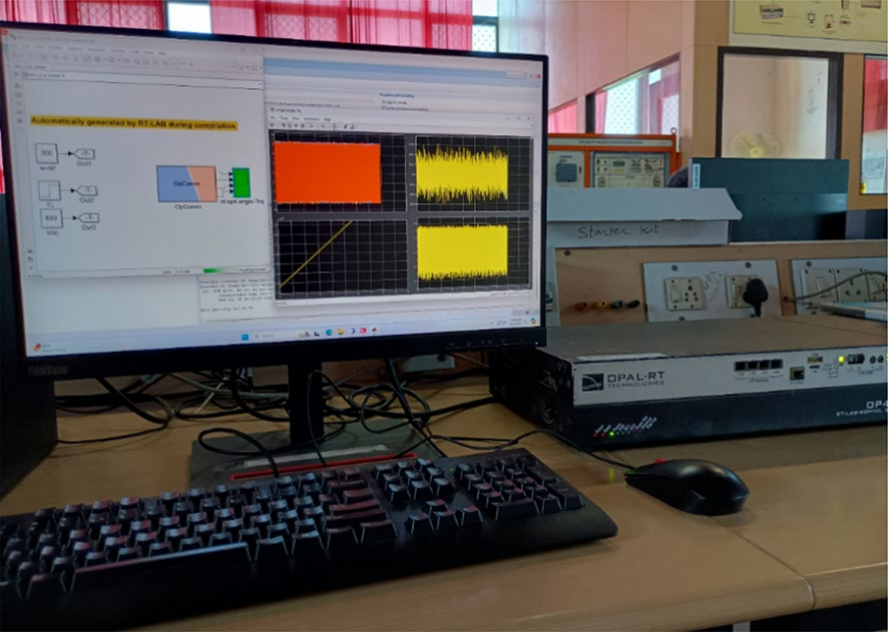
Opal-RT physical setup.

**Fig. 23:**
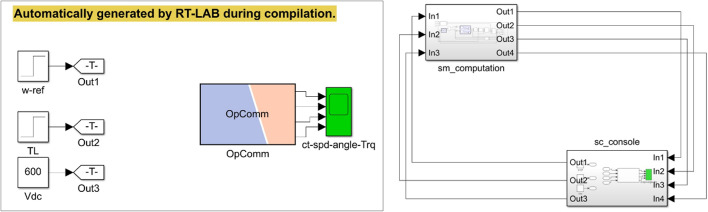
Opal-RT model.

**Fig. 24:**
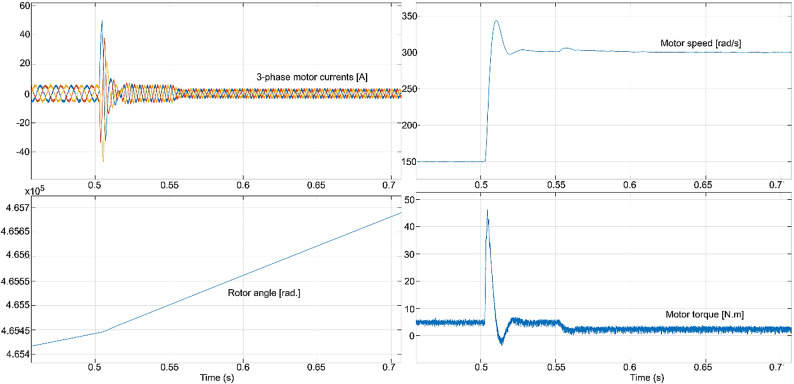
PI controller : current, speed, rotor angle and torque responses.

**Fig. 25:**
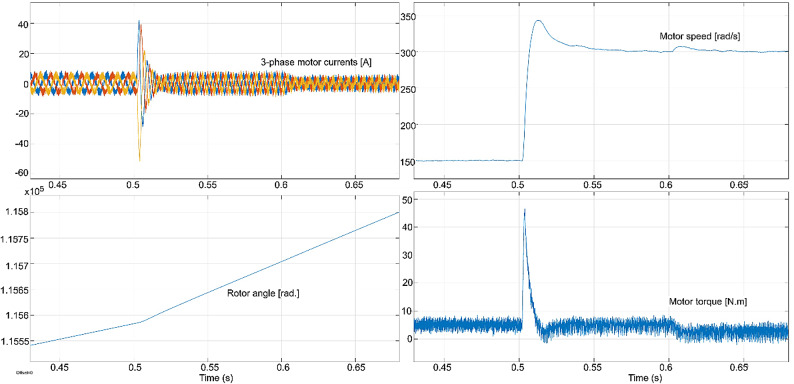
LR controller : current, speed, rotor angle and torque responses.

**Fig. 26:**
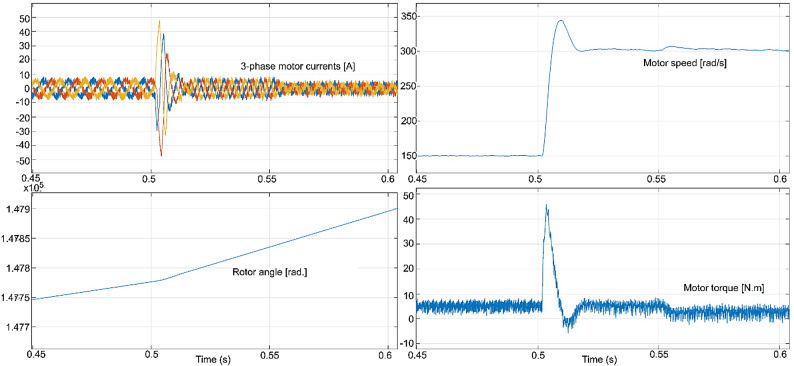
SVM controller : current, speed, rotor angle and torque responses.

**Fig. 27:**
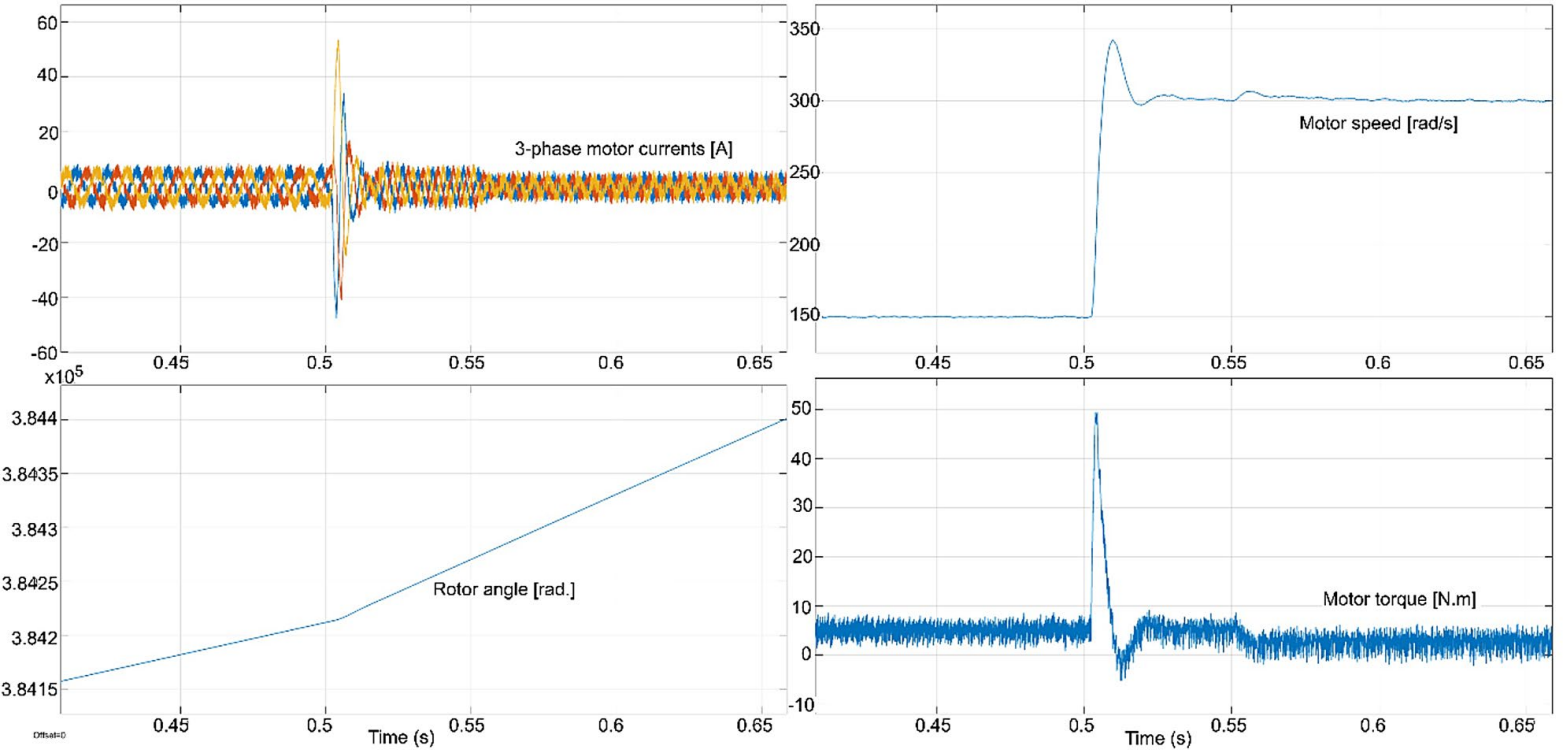
NN controller : current, speed, rotor angle and torque responses.

The conventional and proposed ML-based controllers are tested using Software-in-loop (SIL) mode on the OpalRT device OP4500. Figure 22 gives the Opal-RT setup on SIL mode with OP4500 device. Figure 23 gives the simulation model used to test all the controllers. The ML models have been designed manually using data from the trained models of MATLAB Code. Figures 24, 25, 26 and 27 show the speed and torque responses from the OP4500 device, for the conventional PI controller and the proposed ML-based (LR, SVM, NN) controllers at varying speeds and load conditions. The starting speed is set at 150 rad/s and increased to 300 rad/s at 0.5s timestep. The motor is started on full load and reduced to half load at 0.55s timestep. Compared to the PI controller, the LR-based controller has lesser speed and torque transients when the speed changes. The SVM and NN-based controllers give similar responses as the PI controller.

**Fig. 28:**
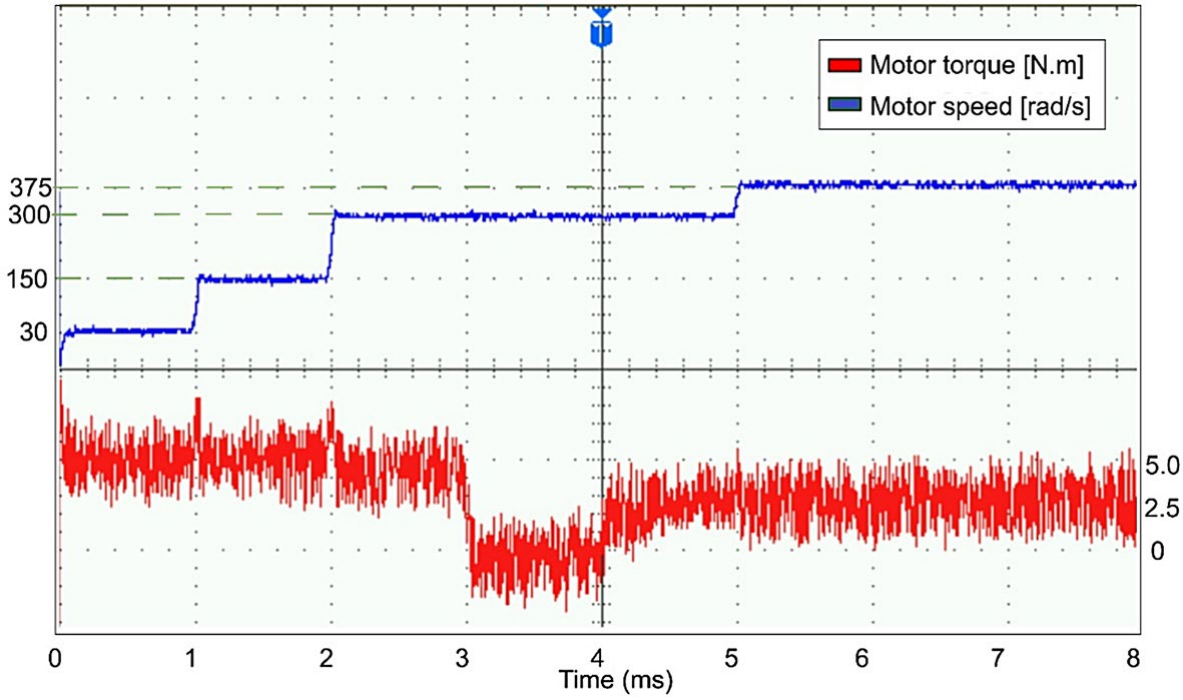
PI controller : speed and torque responses.

**Fig. 29:**
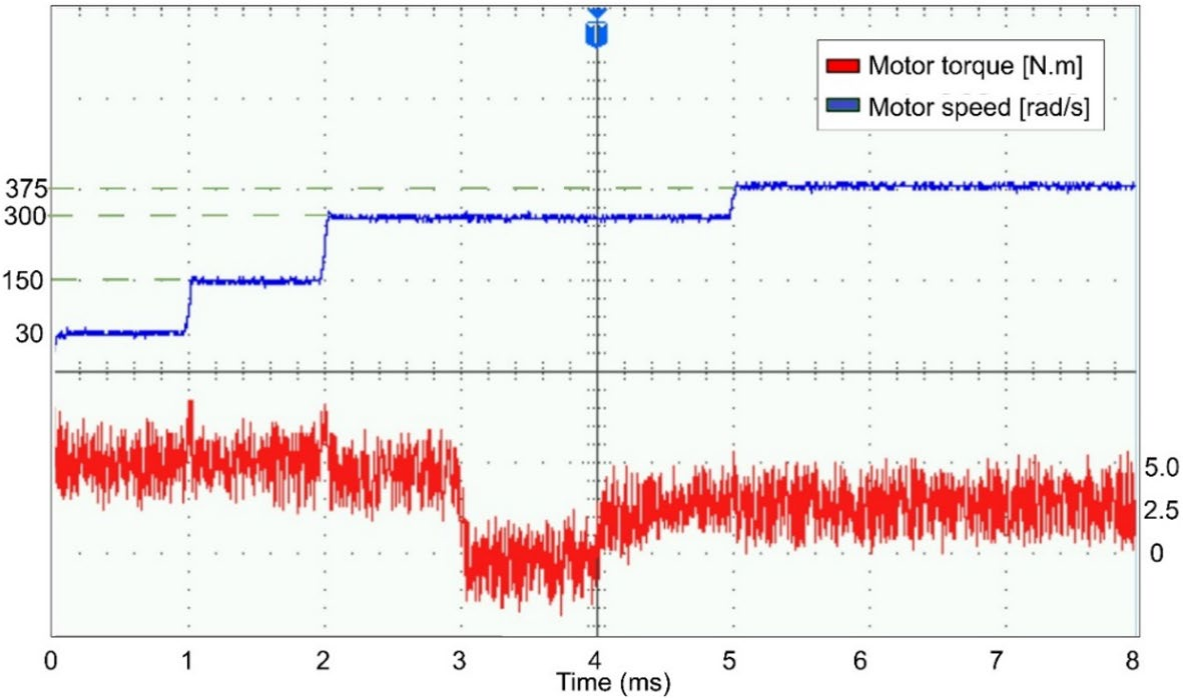
LR controller : speed and torque responses.

**Fig. 30:**
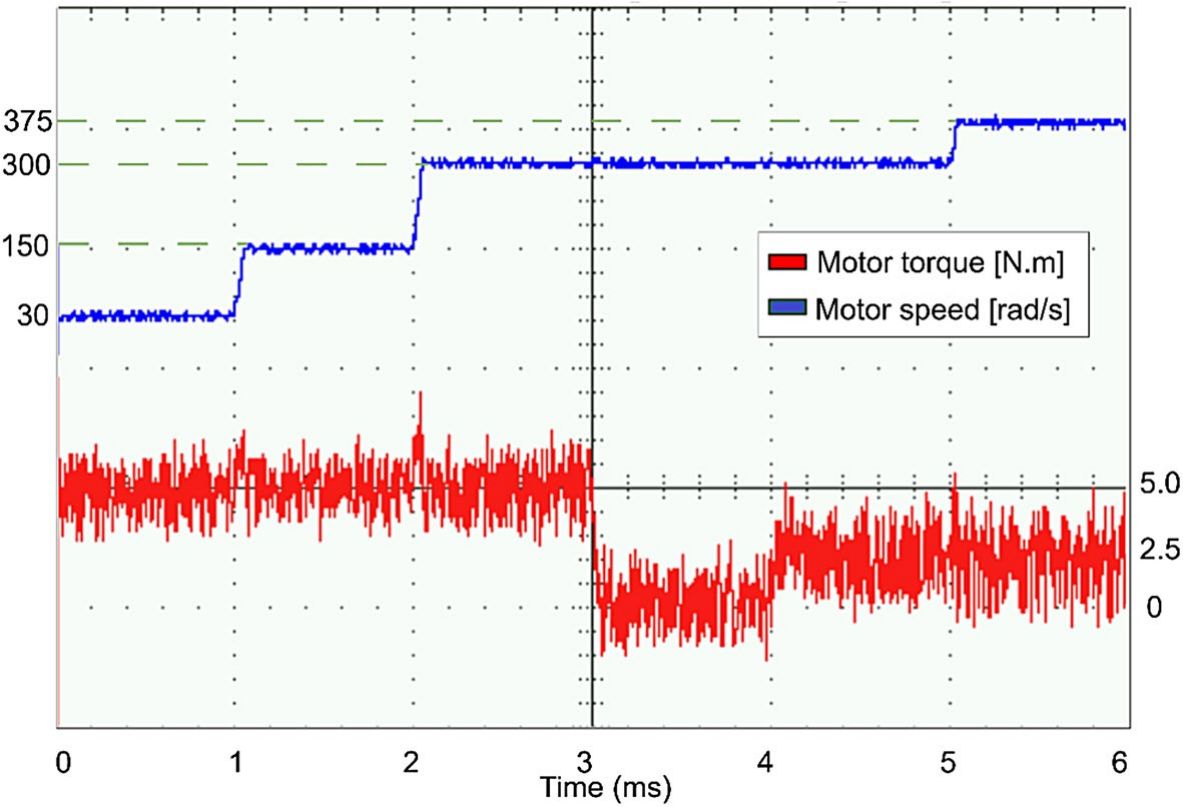
SVM controller : speed and torque responses.

**Fig. 31:**
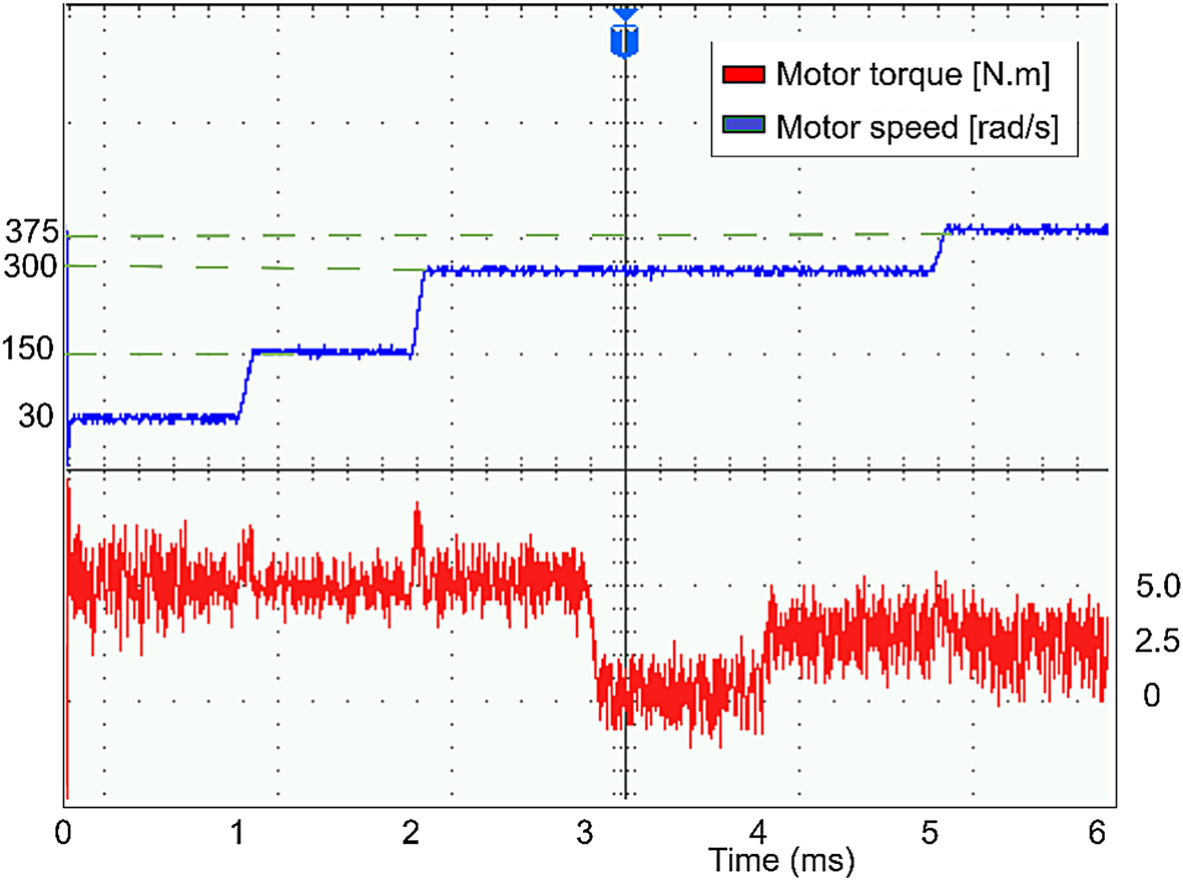
NN controller : speed and torque responses.

Figures 28, 29, 30 and 31 show the results from a digital oscilloscope, for speed variations of 30-150-300–375 rad/s at the time steps 0-1-2-5 ms, and load variations of 5-0-2.5 Nm at the time steps 0-3-4 ms.

## Results & discussion

The conventional and proposed ML-based controllers are validated using MATLAB simulation and Real-Time environment, under different test conditions. The speed and torque response, current response, and dq-axis reference voltages of the drive are considered for evaluation.

### Simulation results

The evaluation of the overall drive response under rated conditions, load disturbances, and speed variations, shows that the responses of the ML-based controllers (LR, SVM, and NN) resemble those of the PI controller, even in the absence of additional circuitry. The graphs make it evident that for all the speeds (below and above the rated speed), the LR-based controller is a good alternative to the PI controller. This is supported by the performance metrics in Table 5, where the LR-based controller gives the least error values, in all the test scenarios. SVM and NN-based controllers give the same metric values as the PI controller.

In the case of speed transients (from Fig. 10), the peak overshoot of the LR-based controller is only 1.138% compared to 1.15% of the PI controller. The SVM and NN-based controllers also give lesser overshoot compared to the PI controller (1.147% and 1.149% respectively). From the speed response of Fig. 15, the LR-based controller has lesser overshoot and hence better performance above half-speed. As seen in Fig. 19, any of the proposed LR, SVM, NN-based controllers can easily reproduce the speed performance of the conventional controller in the case of a load disturbance. From the current response in Fig. 13, motor currents of the LR-based controller can be seen as settling faster (0.012s) compared to the other controllers (0.02s), i.e. 40% faster response. In the case of the NN-based controller, it replicates the performance of the PI controller at all speed levels.

When it comes to testing the robustness of the proposed controllers, Fig. 20 shows that the ML-based (LR, SVM, NN) controllers are capable of giving the same performance as the PI controller, even with increased mechanical parameters.

One main limitation is the low-speed performance of the LR and SVM-based controllers. As seen in Fig. 15, below half-speed, they have slightly higher overshoots compared to the PI controller. For the LR-based controller, the overshoot is less, above half-speed. For the SVM-based controller, the overshoot is lesser only around half-speed. The LR-based controller gives better performance than the PI controller above half-speed conditions, while the NN-based controller gives the same performance as the PI controller.

The performance of the LSTM-based controller was not found satisfactory under different running conditions, when compared to the other controllers.

### Real-time results

Evaluating the controllers in a SIL environment with a physical real-time device, the LR-based controller has lesser speed transients during speed change but is slower in settling back to the desired speed after a load change is applied, as compared to the PI controller response. SVM and NN-based controllers do not perform any better or worse, when compared to the PI controller. LR-based controller needed 0.04s while the PI controller required 0.015s to regain the desired speed after a change in load from no-load to full-load. During speed change from 10 to 100% of the rated speed, the PI controller gives an overshoot of 1.26% while the LR-based controller has only 1.06% overshoot.

**Table 6:** Comparison with existing work.

Ref.s	Paper highlights	Control system	current controller type	External addition of Decoupling terms	Runtime variations in decoupling terms accounted for
1	Unified FOC controller for PMSM	FOC	PI	Yes	Yes
5	Adaptive PID speed control	FOC	Adaptive PID	Yes	Yes
6	Modified reference currents	IFOC	PI	Yes	Yes
8	PMSM drive	VC	Hysteresis & PWM current controllers	Yes	Yes
9	Brain emotional controller (BEC) for speed control	IFOC	PI	No	No
11	Vector control and hall-effect sensors	IFOC	PI	No	No
13	BEC for current control	IFOC	BEC	No	No
15	PMSM Vector control eTPU function	VC	PI	Yes	Yes
29	NN-based current controller	IFOC	NN	No	No
32	Integrated Electric Drive System for Electric Vehicle	IFOC	PI	Yes	Yes
48	ML-based current controller	IFOC	LR/SVM/NN	No	No
**Proposed**	**ML-based current controller**	**IFOC**	**LR/SVM/NN/LSTM**	**No**	**Yes**

### Comparison with existing work

In the conventional vector control scheme, the compensation terms (-*w*_***e***_*L*_***q***_*i*_***q***_, *w*_***e***_*L*_***d***_*i*_***d***_, *w*_***e***_*Ψ*_***f***_) are added to the PI controller outputs externally to avoid decoupling inaccuracy. The constant presence of compensation terms which are time-dependent functions of the dq-axis motor currents and motor speed, has a large impact on the dq- reference voltage signals. In this work, their constant presence is acknowledged. The training data set for the ML models is inclusive of the compensation terms (Fig. 7). The ML models are designed such that the controller outputs given to the next stage in the drive are independent of the external addition of the compensation terms. In other words, the ML models have the compensation terms in-built, in comparison to the conventional PI controller setup. This allows for reduced circuitry in the proposed technique. The proposed ML-based controllers merely need a dataset (inclusive of various scenarios) from an existing technique to prepare the controller for any situation, whereas the conventional controller requires precise PI tuning and occasionally re-tuning in case of sudden system uncertainties. Table 6 gives a comparison of the proposed work with some of the existing literature.

**Fig. 32:**
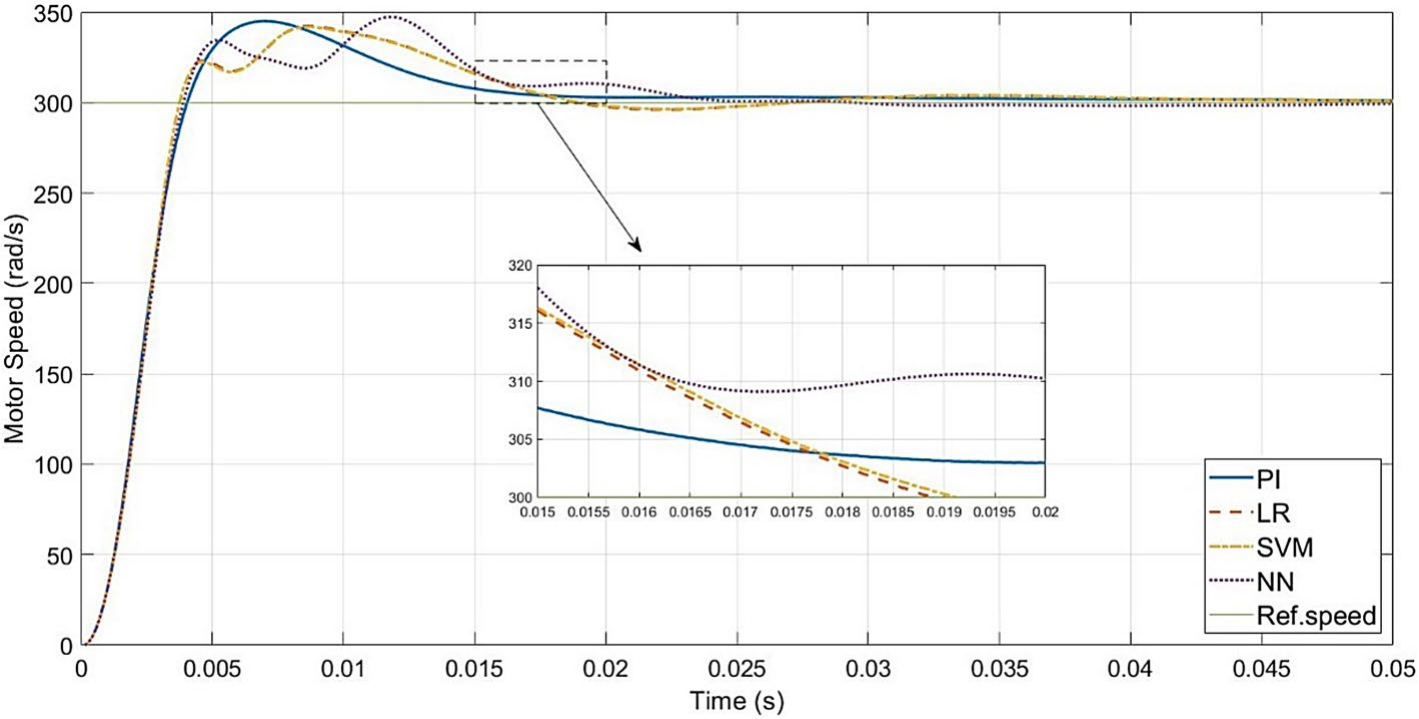
ML-based vs. conventional controllers: Speed response of the drive^48^.

**Fig. 33:**
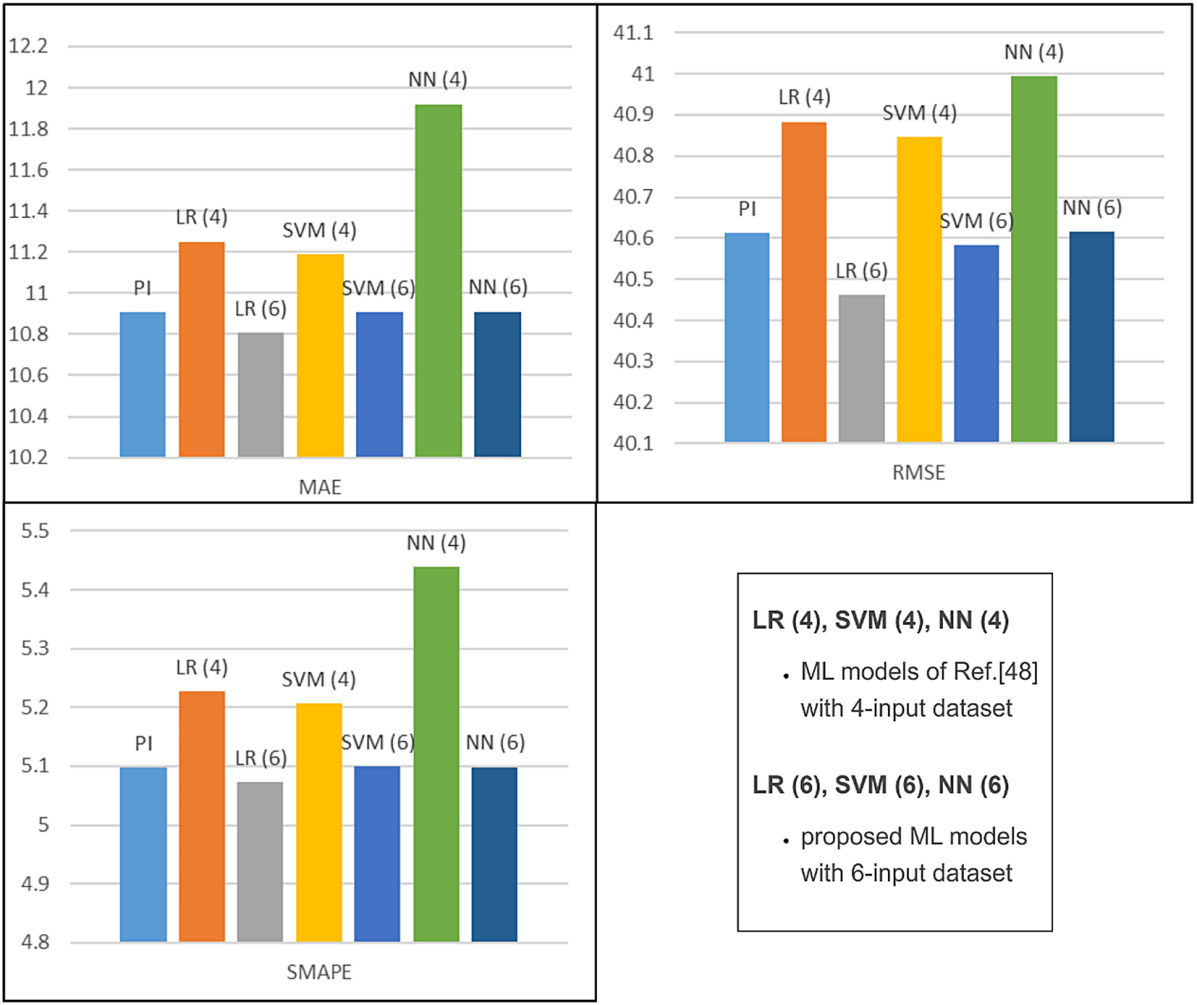
Comparison of evaluation metrics for speed response (Table 7).

Ref.^29^ uses a strategy similar to the proposed work of this paper, with a NN-based current controller. The input dataset, however, does not account for the constant feedback of the compensation terms, which are time-varying parameters. As demonstrated in Ref.^48^ where the technique was investigated for LR, SVM, and NN-based controllers, this has an impact on the final output of the motor drive, in the case of ML-based controllers. Figure 32 shows the speed response of Ref.^48^ at rated conditions. Table 7 gives a comparison between the speed responses of Ref.^48^ and the proposed work, in the same environment. It highlights a reduction in the evaluation metrics, peak overshoot and settling times of the speed responses of the ML-based controllers, when the decoupling terms are included in the dataset and during actual operation. This is also highlighted by Fig. 33. The lowest MAE, SMAPE and RMSE values are given by LR model with 6 inputs, in comparison to PI controller and other ML-based models with 4 inputs or 6 inputs.

A network with two hidden layers, each including six neurons, is used in the NN model of Ref^29^. In contrast, when the decoupling terms are added as inputs to the NN model, the proposed NN model in this work only needs one hidden layer and two neurons. Because of this, the NN model that this paper proposes is more compact than the one that already exists. Table 8 gives comparison of proposed NN model with the existing models of Ref.s^29] and [48^.

In addition to challenging the conventional controller and the work in Ref.^29^ in terms of the input dataset, this paper also examines other ML-based models which are not yet been explored for current control.

**Table 7:** Comparison of speed response at rated conditions [Fig.s 10 & 32].

	Features	PI	LR	SVM	NN
48	MAE	**10.9072**	11.2512	11.1903	11.9194
	RMSE	**40.6125**	40.8831	40.8455	40.9932
	SMAPE	**5.0976**	5.2276	5.2072	5.4389
	Peak overshoot (%)	1.15	1.14	1.139	1.157
	Settling time (s)	0.018	0.02	0.02	0.022
Proposed	MAE	10.9072	**10.8093**	10.9081	10.9096
	RMSE	40.6125	**40.4614**	40.5824	40.6145
	SMAPE	5.0976	**5.0724**	5.0998	5.0987
	Peak overshoot (%)	1.15	1.138	1.147	1.149
	Settling time (s)	0.018	0.018	0.018	0.018

**Table 8:** Comparison of NN models.

Features	[29]	[48]	Proposed
No. of hidden layers	2	2	1
Size of hidden layers	6 + 6	10 + 2	2
No. of input variables	4	4	6
No. of output variables	2	2	2
Activation function (hidden layer)	tanh( )	tansig( )	tansig( )
Training algorithm	Levenberg-Marquardt	Levenberg-Marquardt	Levenberg-Marquardt

## Conclusion

This paper presents different ML-based speed control algorithms for a vector-controlled PMSM drive. The main objective is to explore the feasibility of applying machine learning in the speed control of motor drives. Under various test scenarios, the performances of the conventional PI controller and the proposed ML-based controllers are studied. In comparison to the conventional controller that requires compensation term feedback, the proposed ML-based controllers can achieve better performance without the additional feedback circuitry.

The performance metrics of the LR-based controllers were found to be better than those of the conventional PI controller. In every test situation, the errors of the LR-based controller are lesser by a margin of up to 1.86%. While maintaining the same settling time for the speed and torque waveforms, the transient peaks are lesser for the LR-based controller in most cases by 0.012% and a 40% reduction in current response time. The SVM-based and NN-based controllers give a 0.003% reduction in the transient peaks of the responses and a 0.48% improvement in the metrics, compared to the conventional controller. Their performance metrics and responses are on par with those provided by the PI controller. The LSTM-based controller was found to be in need of more training for better performance.

By fine-tuning the training parameters and using a larger data set, the performance of all the proposed ML-based controllers can be further enhanced.

## Data Availability

The datasets used and/or analysed during the current study available from the corresponding author on reasonable request.

## References

[1] Koç, M. Unified Field Oriented Controlled Drive System for All Types of PMSMs Considering System Nonlinearities, in *IEEE Access*, vol. 10, pp. 56773–56784, (2022).

[2] Kolano, K. New Method of Vector Control in PMSM Motors, in *IEEE Access*, vol. 11, pp. 43882–43890, (2023).

[3] Khanh, P. Q. & Anh, H. P. H. Advanced PMSM speed control using fuzzy PI method for hybrid power control technique. in *Ain Shams Eng. Journal*, **14**, 12, (2023).

[4] Madichetty, S., Mishra, S. & Basu, M. New trends in electric motors and selection for electric vehicle propulsion systems, *IET Electr. Syst. Transp*., vol. 11, no. 3, pp. 186–199, Sep. (2021).

[5] Jung, J. W., Leu, V. Q., Do, T. D., Kim, E. K. & Choi, H. H. Adaptive PID speed control design for permanent magnet synchronous motor drives. *IEEE Trans. Power Electron.* **30** (2), 900–908 (Feb. 2015).

[6] Soliman, H. M. Improve the performance characteristics of the IPMSM under the effect of the varying loads. *IET Electr. Power Appl.* **13** (12), pp1935–1945 (2019).

[7] Rind, S. J., Ren, Y., Hu, Y., Wang, J. & Jiang, L. Configurations and control of traction motors for electric vehicles: A review. *Chin. J. Electr. Eng.* **3** (3), 1–17 (December 2017).

[8] Pillay, P. & Krishnan, R. Modeling, simulation, and analysis of permanent-magnet motor drives. I. The permanent-magnet synchronous motor drive. in *IEEE Trans. Ind. Applications*, **25**, 2, pp. 265–273, March-April 1989.

[9] Qutubuddin, M. D. & Yadaiah, N. A new intelligent adaptive mechanism for sensorless control of permanent magnet synchronous motor drive. *Biologically Inspired Cogn. Architectures*. **24**, 47–58 (2018).

[10] Raia, M. R., Ruba, M., Nemes, R. O. & Martis, C. Artificial Neural Network and Data Dimensionality Reduction Based on Machine Learning Methods for PMSM Model Order Reduction, in *IEEE Access*, vol. 9, pp. 102345–102354, (2021).

[11] Sorial, R. R., Soliman, M. H., Hasanien, H. M. & Talaat, H. E. A. A Vector Controlled Drive System for Electrically Power Assisted Steering Using Hall-Effect Sensors, in *IEEE Access*, vol. 9, pp. 116485–116499, (2021).

[12] Hannan, M. A. et al. Switching Techniques and Intelligent Controllers for Induction Motor Drive: Issues and Recommendations, in *IEEE Access*, vol. 6, pp. 47489–47510, (2018).

[13] Qutubuddin, M. D. & Yadaiah, N. Modeling and implementation of brain emotional controller for permanent magnet synchronous motor drive. *Eng. Appl. Artif. Intell.* **60**, 193–203 (2017).

[14] Liu, C. & Luo, Y. Overview of advanced control strategies for electric machines. *Chin. J. Electr. Eng.* **3** (2), 53–61 (September 2017).

[15] Brejl, M. & Princ, M. Using the PMSM Vector Control, Freescale Semiconductor, 2012 [Online]. Available: https://www.nxp.com/docs/en/application-note/AN2972.pdf Accessed on: Jun.5, 2022.

[16] Kulkarni, P. Sensorless Field-Oriented Control of Permanent Magnet Synchronous Motor (Surface and Interior) for Appliances with Angle-Tracking Phase-Locked Loop Estimator, Microchip Technology Inc., [Online] (2019). Available: https://ww1.microchip.com/downloads/en/DeviceDoc/TB3220-Sensorless-Field-Oriented-Control-of-PMSM-for-Appliances-DS90003220A.pdf Accessed on: July 16,2024.

[17] Infineon Technologies, A. G. Sensorless field-oriented control (FOC) usingpsoc™ 6 MCU, [Online]. (2022). https://www.infineon.com/dgdl/Infineon-AN235096_Sensorless_field-oriented_control_FOC_using_PSoC_6_MCU-ApplicationNotes-v01_00-EN.pdf?fileId=8ac78c8c821f280601821f2bea270000 Accessed on: July 11,2024.

[18] Semiconductors, N. X. P. MCUXpresso SDK Field-Oriented Control (FOC) of 3-Phase PMSM and BLDC Motors - Userguide, 2023 [Online]. Available: https://www.nxp.com/docs/en/user-guide/PMSMMCXN9XXEVK.pdf Accessed on: July 16,2024.

[19] Karim, A., Azam, S., Shanmugam, B., Kannoorpatti, K. & Alazab, M. A Comprehensive Survey for Intelligent Spam Email Detection, in *IEEE Access*, vol. 7, pp. 168261–168295, (2019).

[20] Jaffar, F., Farid, T., Sajid, M., Ayaz, Y. & Khan, M. J. Prediction of Drag Force on Vehicles in a Platoon Configuration Using Machine Learning, in *IEEE Access*, vol. 8, pp. 201823–201834, (2020).

[21] Morais, R. M. & Pedro, J. Machine learning models for estimating quality of transmission in DWDM networks, in *Journal of Optical Communications and Networking*, vol. 10, no. 10, pp. D84-D99, Oct. (2018).

[22] Daliya, V. K., Ramesh, T. K. & Ko, S. B. An Optimised Multivariable Regression Model for Predictive Analysis of Diabetic Disease Progression, in *IEEE Access*, vol. 9, pp. 99768–99780, (2021).

[23] Simeone, O. A Very Brief Introduction to Machine Learning With Applications to Communication Systems, in *IEEE Transactions on Cognitive Communications and Networking*, vol. 4, no. 4, pp. 648–664, Dec. (2018).

[24] Dahrouj, H. et al. An Overview of Machine Learning-Based Techniques for Solving Optimization Problems in Communications and Signal Processing, in *IEEE Access*, vol. 9, pp. 74908–74938, (2021).

[25] Mahmud, K. et al. Machine Learning Based PV Power Generation Forecasting in Alice Springs, in *IEEE Access*, vol. 9, pp. 46117–46128, (2021).

[26] Farsi, B., Amayri, M., Bouguila, N. & Eicker, U. On Short-Term Load Forecasting Using Machine Learning Techniques and a Novel Parallel Deep LSTM-CNN Approach, in *IEEE Access*, vol. 9, pp. 31191–31212, (2021).

[27] Méndez, M., Núñez, M. & M.G. and Machine learning algorithms to forecast air quality: a survey. *Artif. Intell. Rev.* **56**, 10031–10066 (2023). 10.1007/s10462-023-10424-4 PMC9933038 36820441

[28] Martínez, V. & Rocha, A. The Golem: A General Data-Driven Model for Oil & Gas Forecasting Based on Recurrent Neural Networks, in *IEEE Access*, vol. 11, pp. 41105–41132, (2023).

[29] Li, S. et al. Neural-Network vector controller for Permanent-Magnet synchronous motor drives: simulated and Hardware-Validated results. *IEEE Trans. Cybernetics*. **50** (7), 3218–3230 (July 2020). 10.1109/TCYB.2019.2897653 30802881

[30] Zhang, S., Wallscheid, O. & Porrmann, M. Machine learning for the control and monitoring of electric machine drives: advances and trends. *IEEE Open. J. Ind. Appl.* **4**, 188–214 (2023).

[31] Butt, C. B. & Rahman, M. A. Intelligent Speed Control of Interior Permanent Magnet Motor Drives Using a Single Untrained Artificial Neuron, in *IEEE Transactions on Industry Applications*, vol. 49, no. 4, pp. 1836–1843, July-Aug. (2013).

[32] Hu, J., Peng, T., Jia, M., Yang, Y. & Guan, Y. Study on Electromechanical Coupling Characteristics of an Integrated Electric Drive System for Electric Vehicle, in *IEEE Access*, vol. 7, pp. 166493–166508, (2019).

[33] Li, L. & Liu, Q. Research on IPMSM Drive System Control Technology for Electric Vehicle Energy Consumption, in *IEEE Access*, vol. 7, pp. 186201–186210, (2019).

[34] Gutiérrez-Gómez, L., Petry, F. & Khadraoui, D. A Comparison Framework of Machine Learning Algorithms for Mixed-Type Variables Datasets: A Case Study on Tire-Performances Prediction, in *IEEE Access*, vol. 8, pp. 214902–214914, (2020).

[35] Alquthami, T., Zulfiqar, M., Kamran, M., Milyani, A. H. & Rasheed, M. B. A Performance Comparison of Machine Learning Algorithms for Load Forecasting in Smart Grid, in *IEEE Access*, vol. 10, pp. 48419–48433, (2022).

[36] Pirbazari, A. M., Sharma, E., Chakravorty, A., Elmenreich, W. & Rong, C. An Ensemble Approach for Multi-Step Ahead Energy Forecasting of Household Communities, in *IEEE Access*, vol. 9, pp. 36218–36240, (2021).

[37] Zhang, C., Zhang, H. & Hu, X. A Contrastive Study of Machine Learning on Funding Evaluation Prediction, in *IEEE Access*, vol. 7, pp. 106307–106315, (2019).

[38] Shalev-Shwartz, S. & Ben-David, S. *Understanding Machine Learning: from Theory To Algorithms* (Cambridge University Press, 2014).

[39] Naz, F. et al. Comparative Analysis of Deep Learning and Statistical Models for Air Pollutants Prediction in Urban Areas, in *IEEE Access*, vol. 11, pp. 64016–64025, (2023).

[40] Ahmadi, A. et al. Long-Term Wind Power Forecasting Using Tree-Based Learning Algorithms, in *IEEE Access*, vol. 8, pp. 151511–151522, (2020).

[41] Jawad, M. et al. Machine Learning Based Cost Effective Electricity Load Forecasting Model Using Correlated Meteorological Parameters, in *IEEE Access*, vol. 8, pp. 146847–146864, (2020).

[42] Bao, Y., Xiong, T. & Hu, Z. Multi-step-ahead time series prediction using multiple-output support vector regression, in *Neurocomputing*, vol. 129, pp. 482–493, (2014).

[43] Duan, J. & Kashima, H. Learning to Rank for Multi-Step Ahead Time-Series Forecasting, in *IEEE Access*, vol. 9, pp. 49372–49386, (2021).

[44] Lemke, C. & Gabrys, B. Meta-learning for time series forecasting and forecast combination, in *Neurocomputing*, vol. 73, pp. 2006–2016, (2010).

[45] Asthana, P., Mishra, S., Gupta, N., Derawi, M. & Kumar, A. Prediction of Student’s Performance With Learning Coefficients Using Regression Based Machine Learning Models, in *IEEE Access*, vol. 11, pp. 72732–72742, (2023).

[46] Varoquaux, G. & Colliot, O. Evaluating machine learning models and their diagnostic value. *Mach. Learn. Brain Disorders Neuromethods*. **197**, 601–630 (2023). 37988512

[47] Li, T., Sun, X., Yang, Z. & Lei, G. Simplified Two-Step model predictive control with fast voltage vector search. in *IEEE Trans. Industrial Electronics*, 10.1109/TIE.2024.3447757

[48] Tom, A. M. & Febin Daya, J. L. Machine learning techniques for vector control of permanent magnet synchronous motor drives. *Cogent Engineering*, **11**, 1, (2024).

